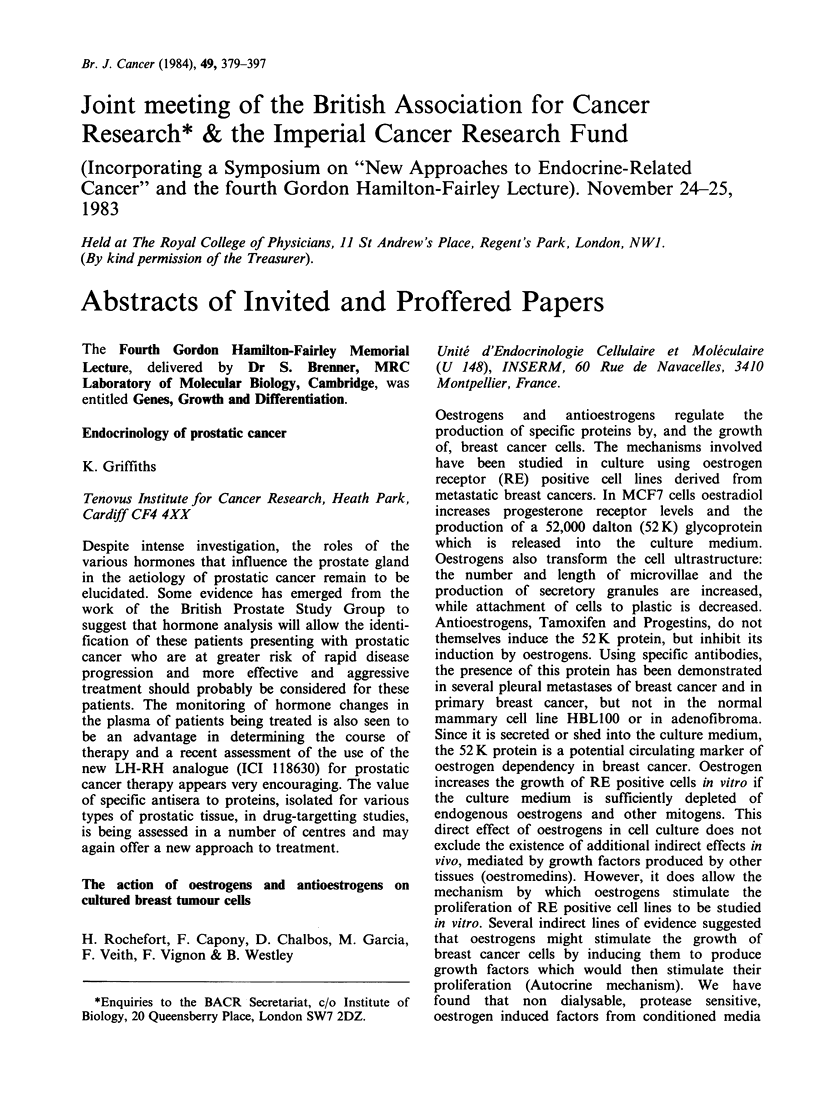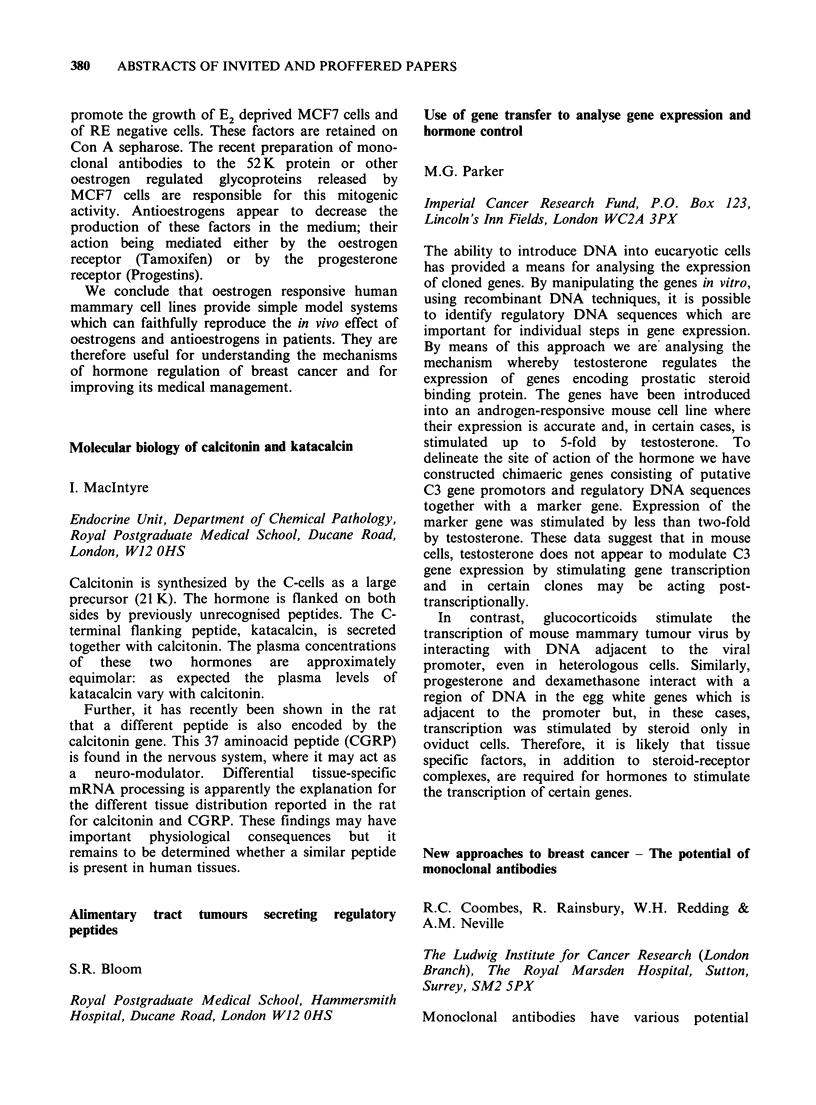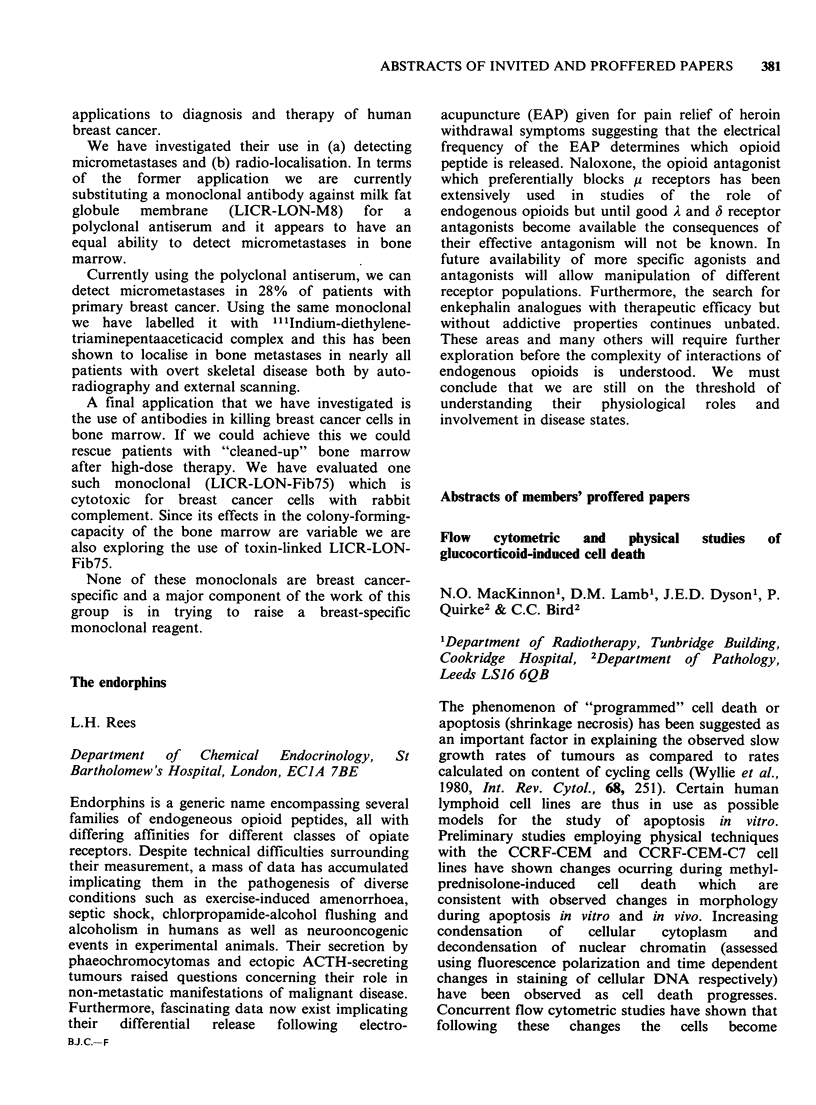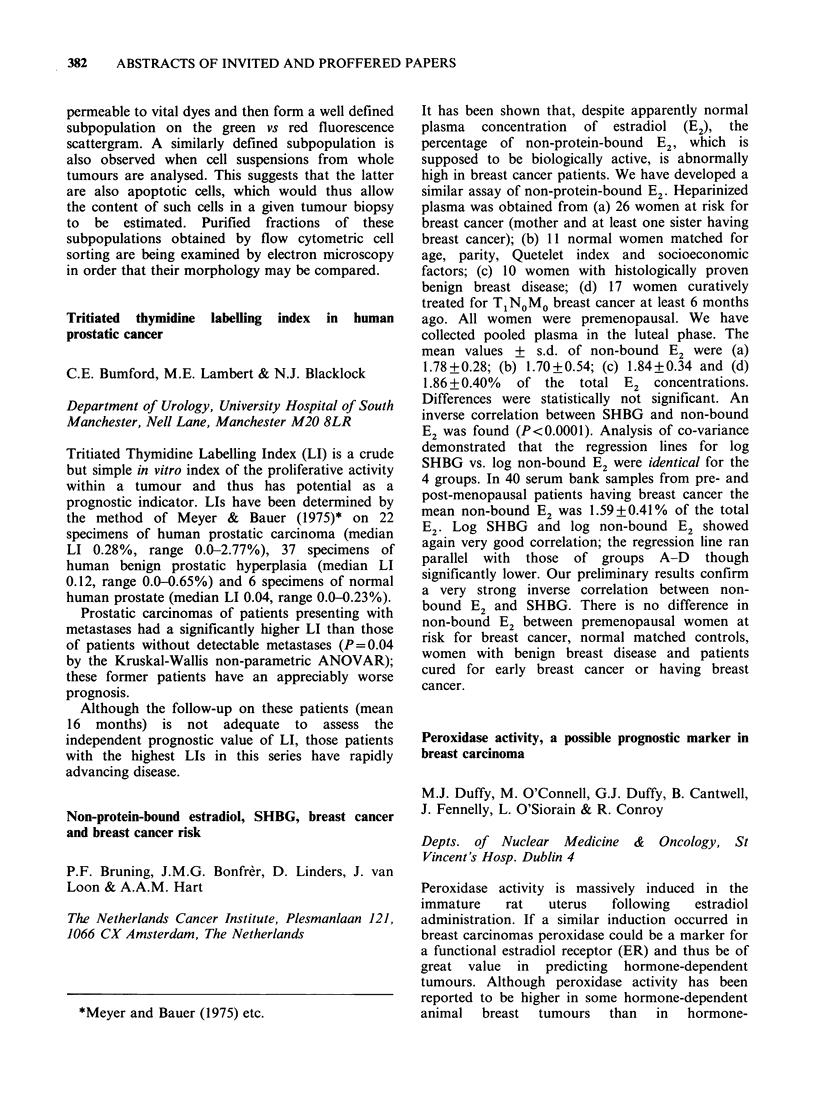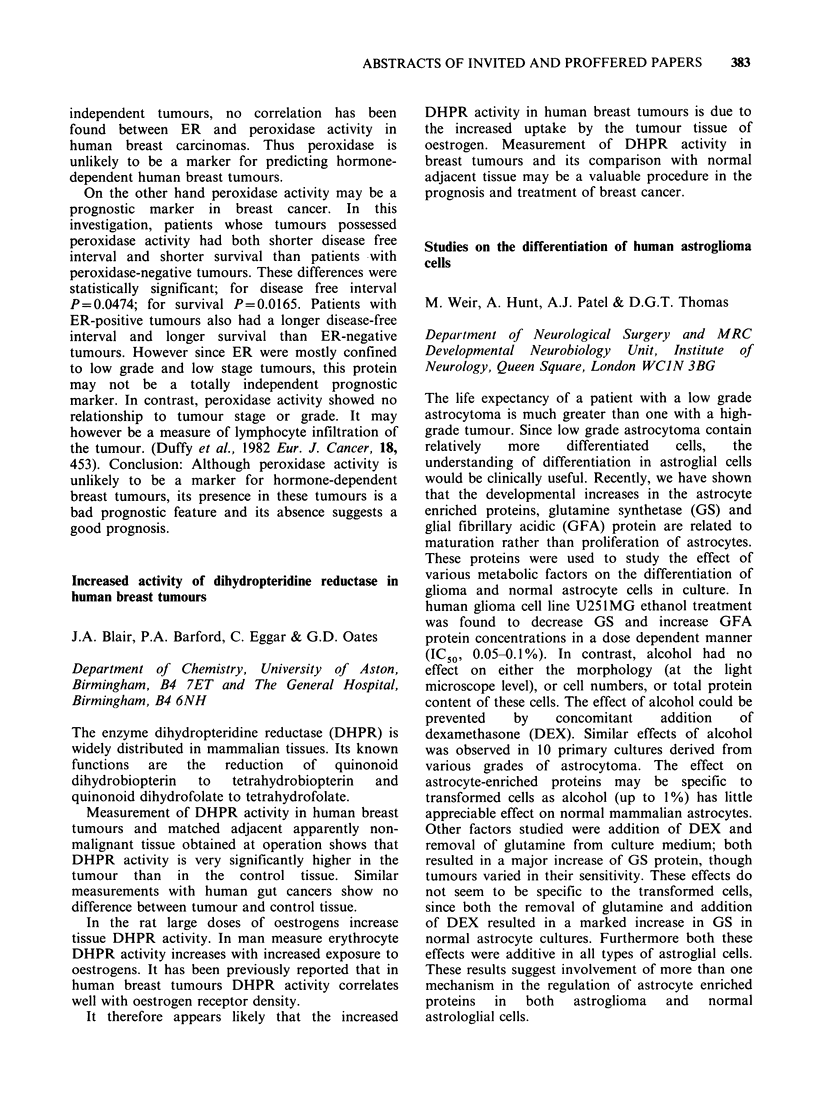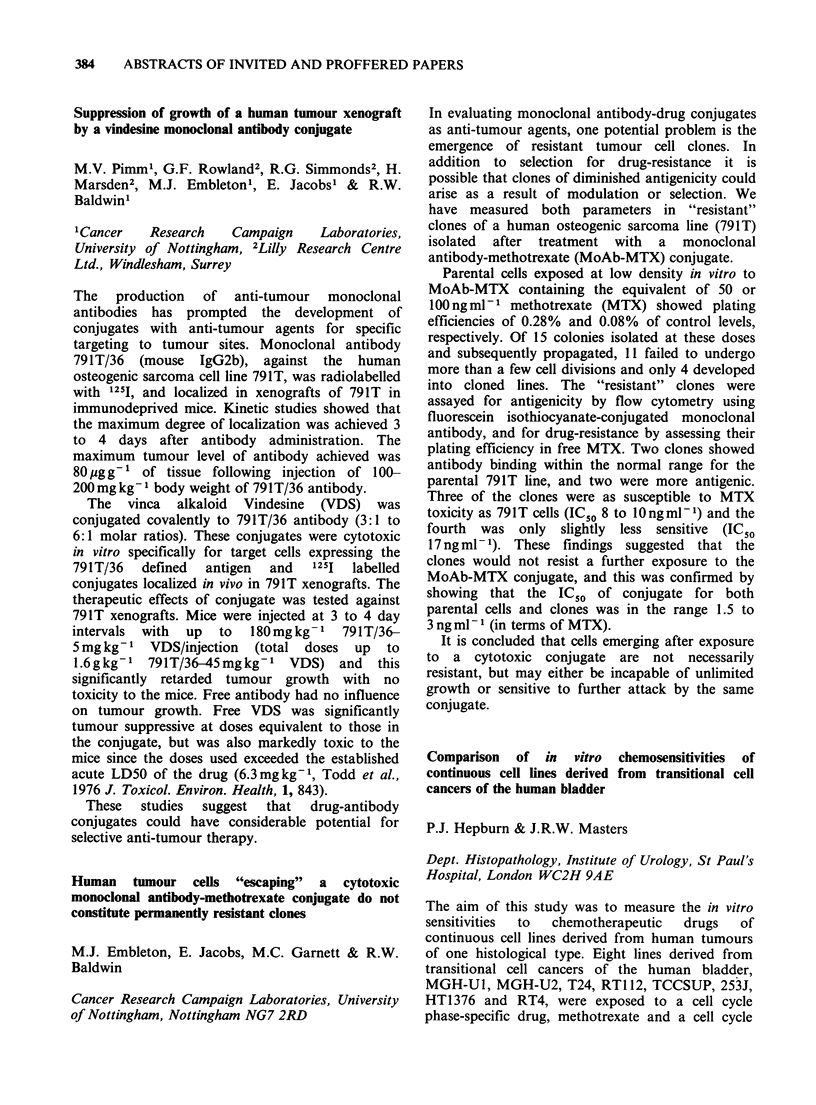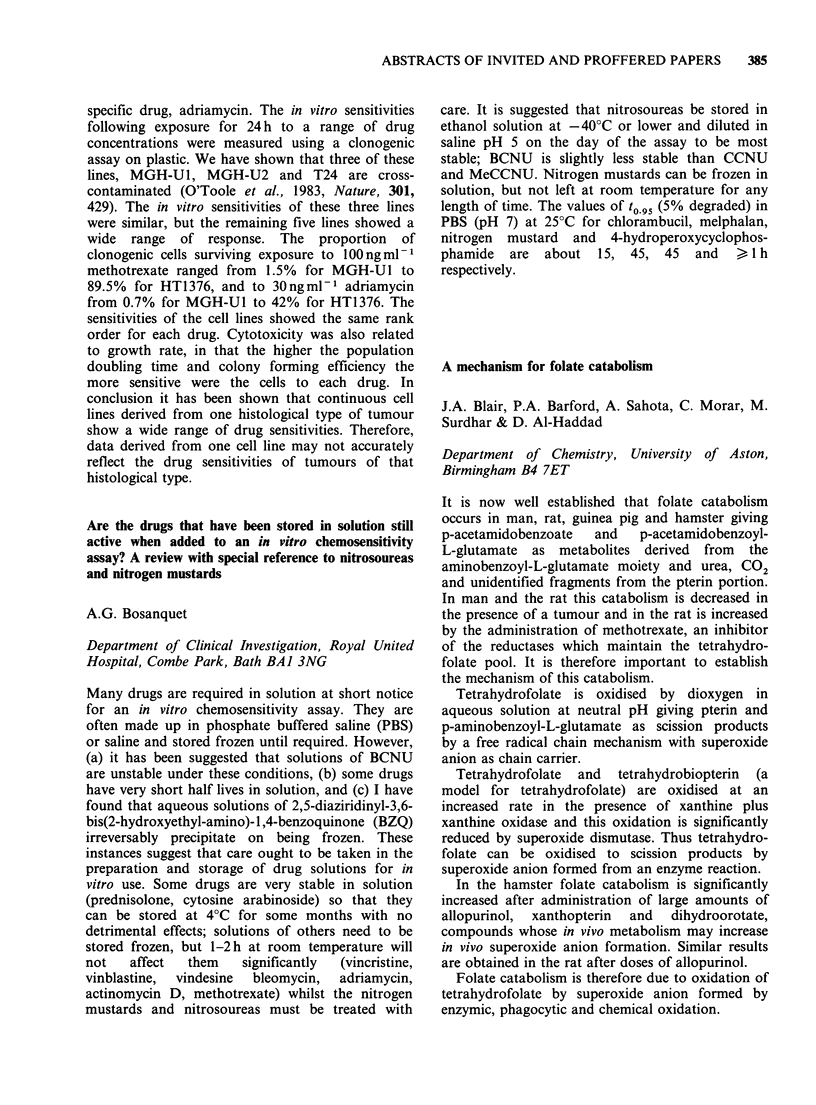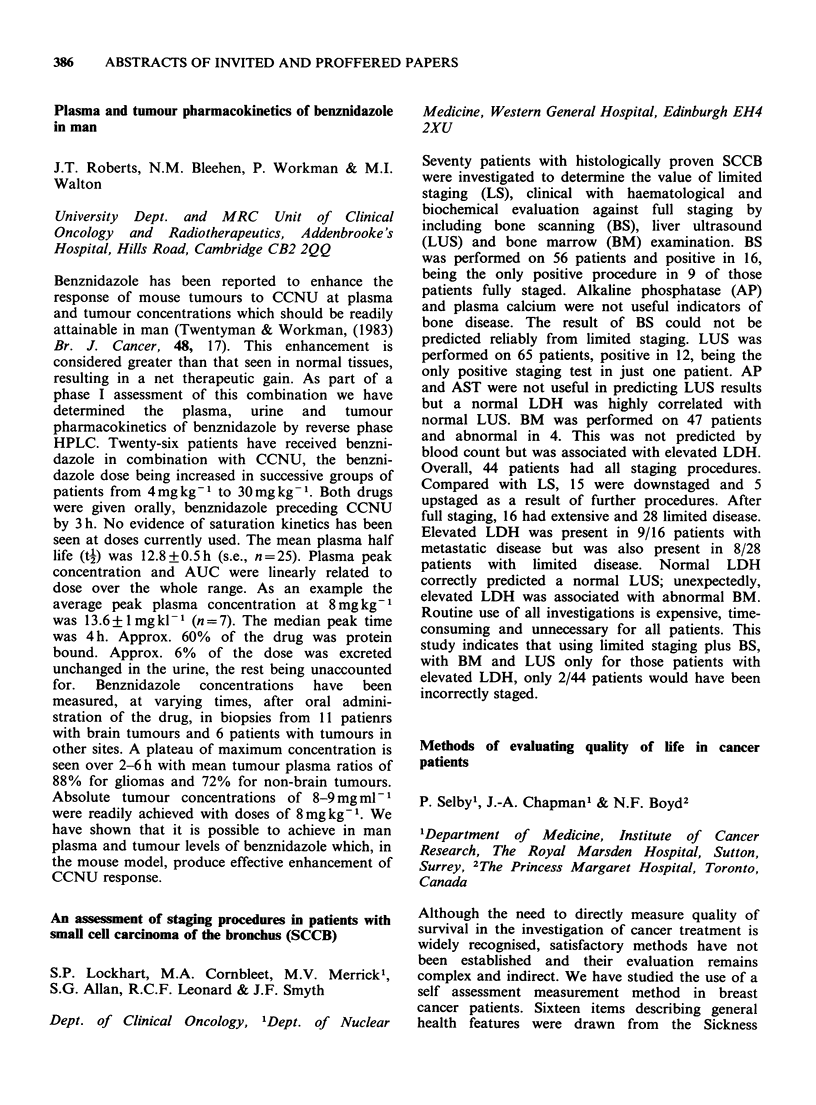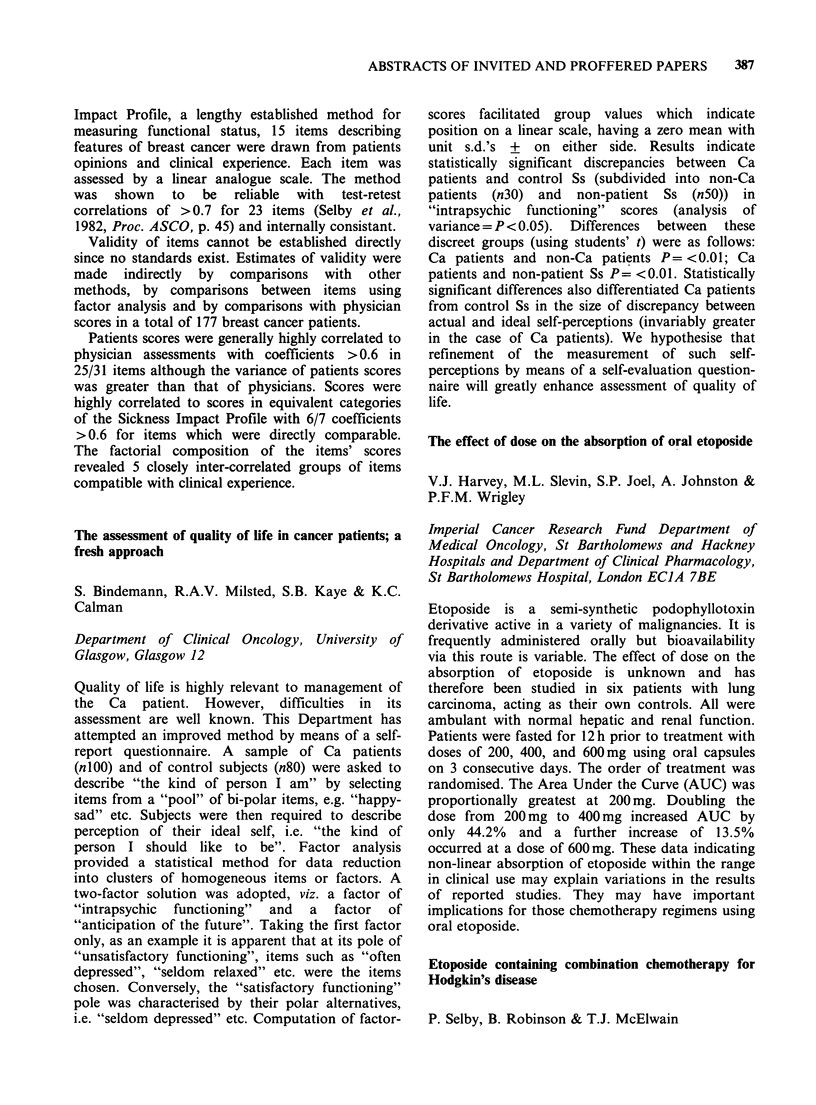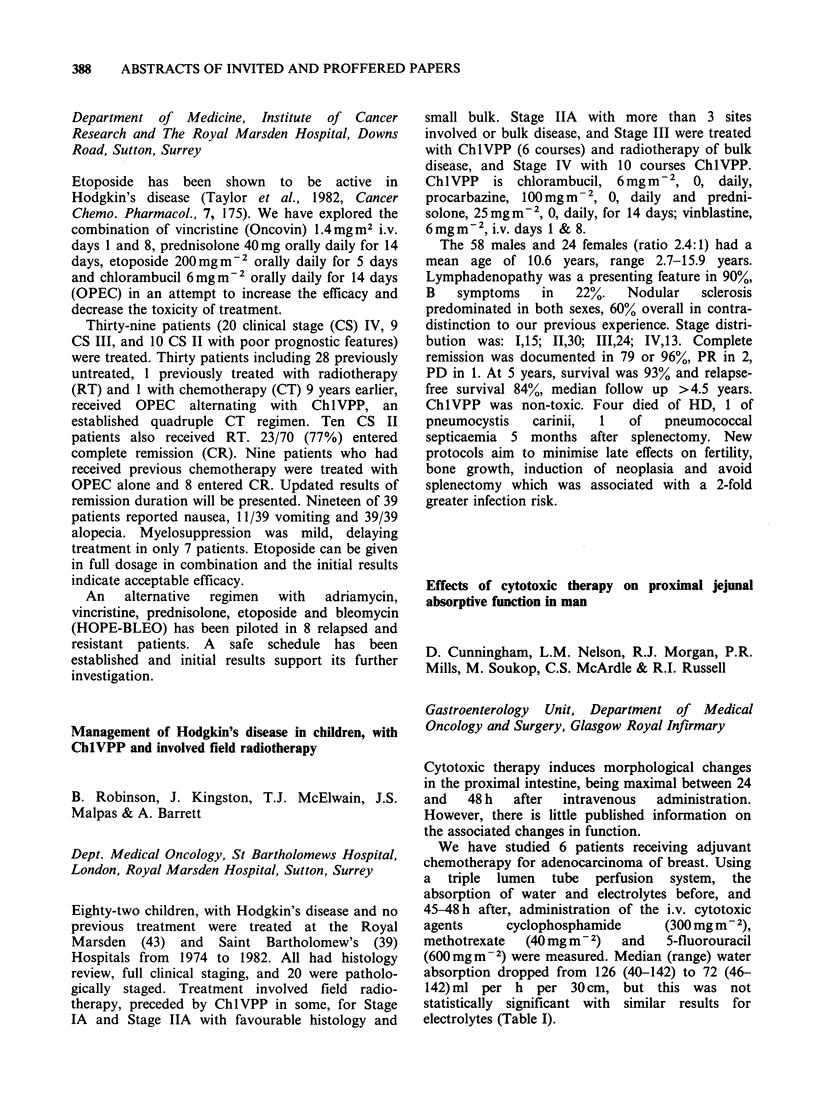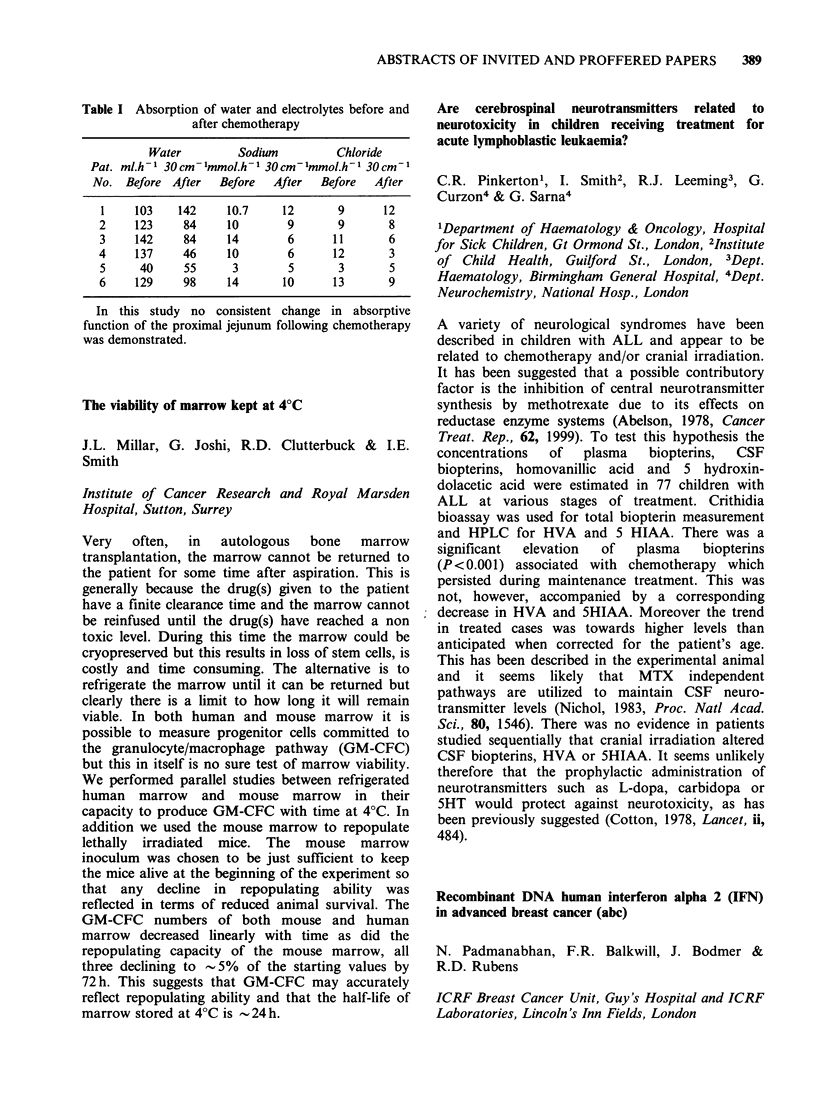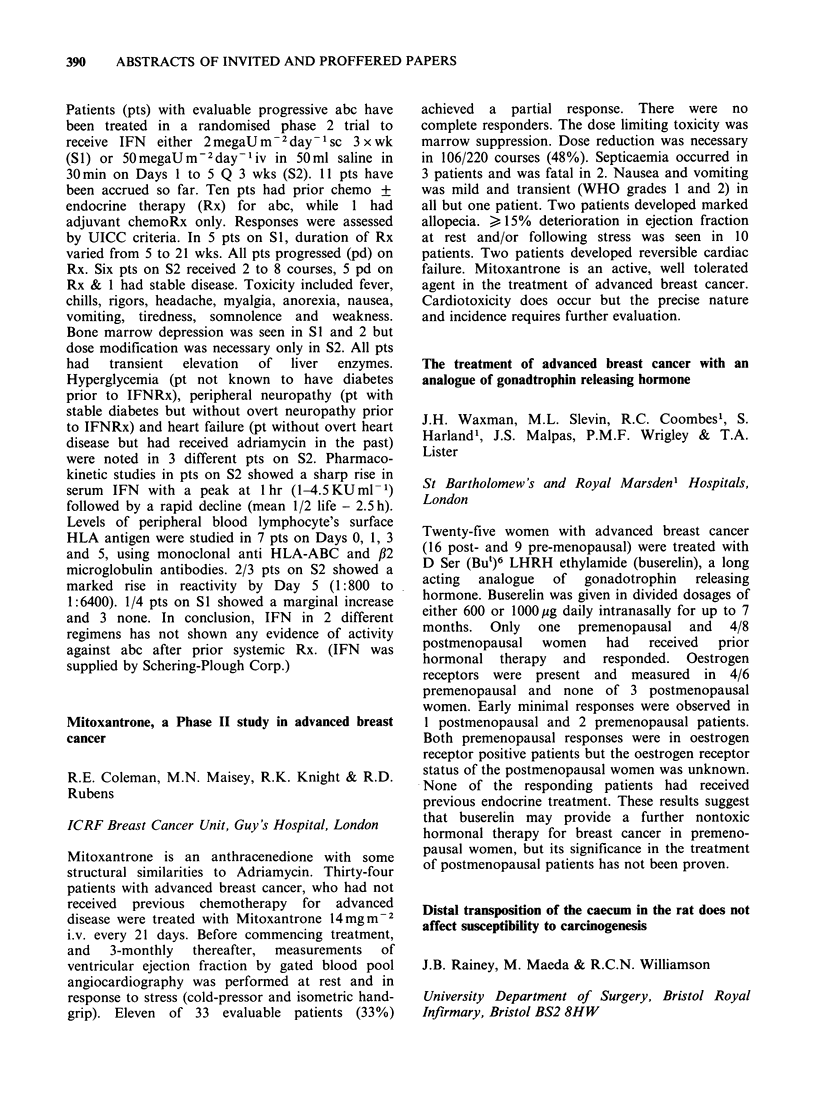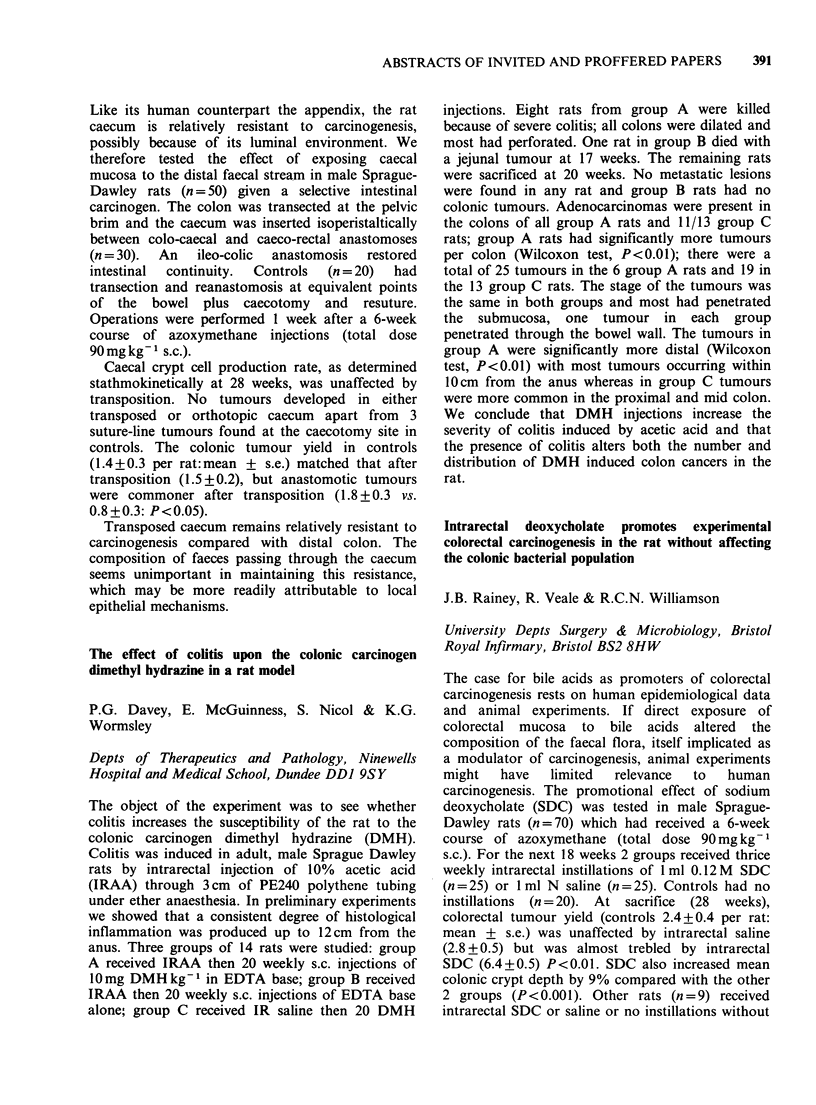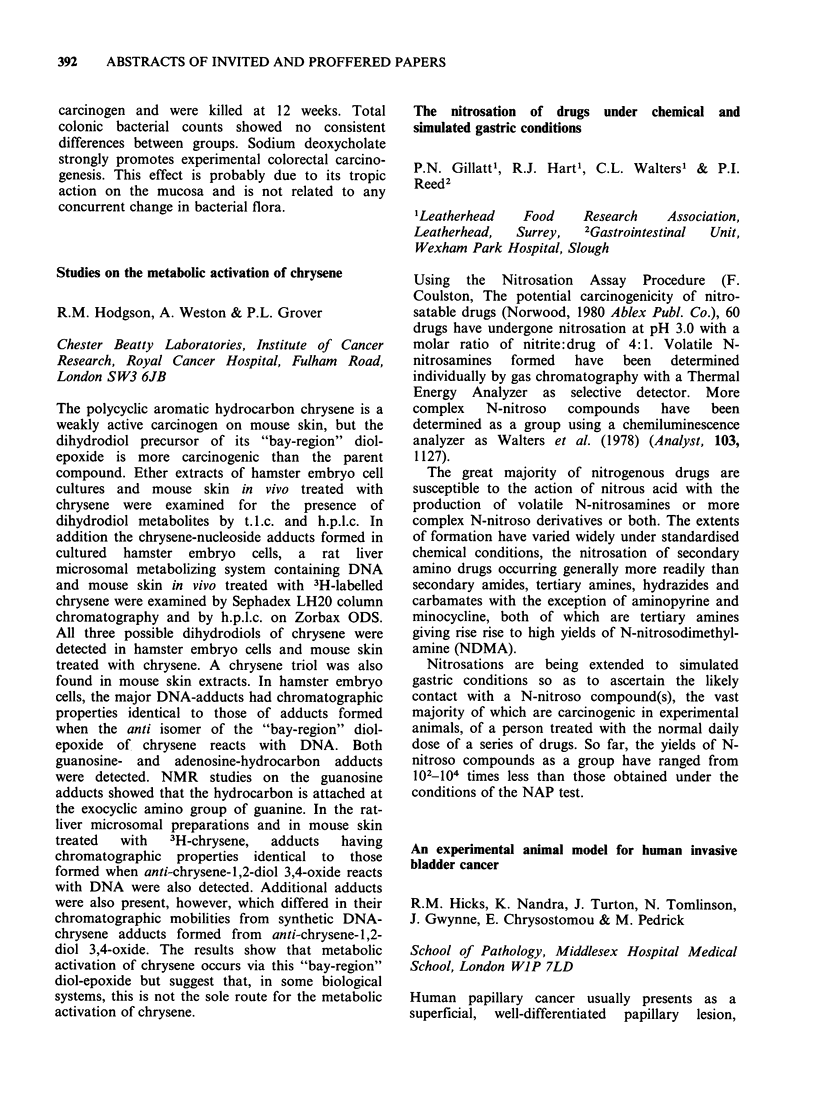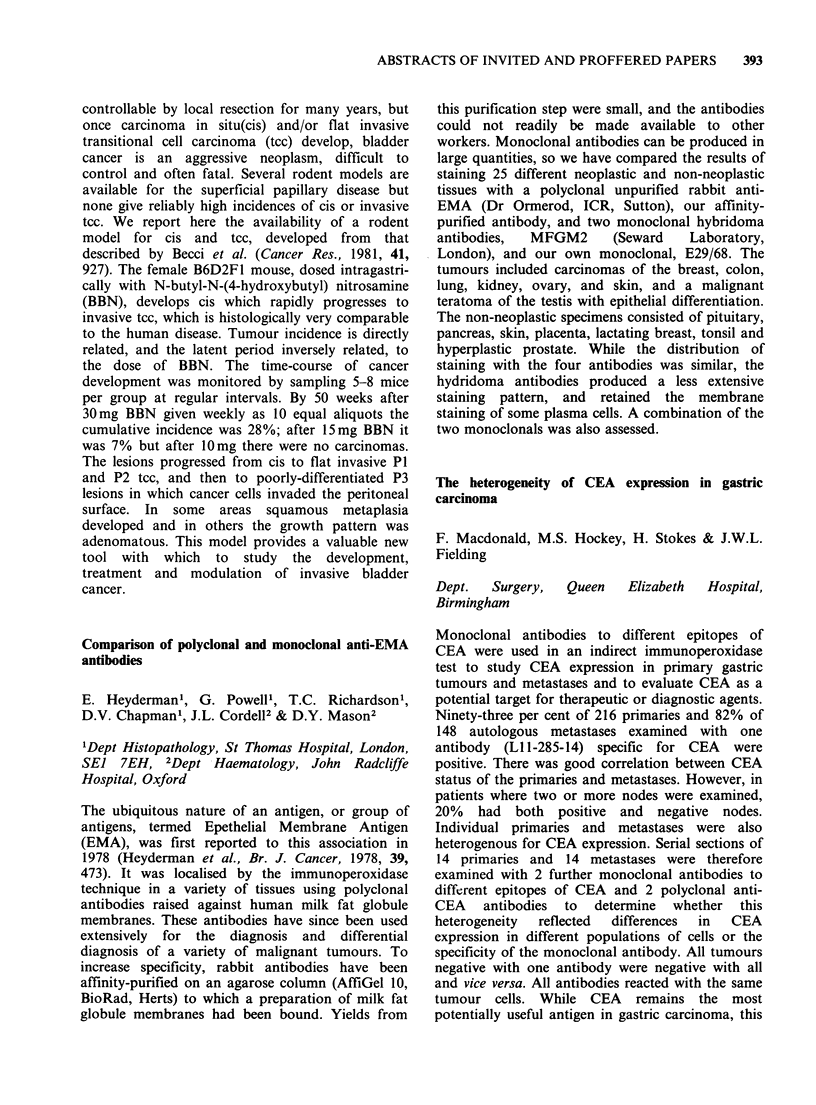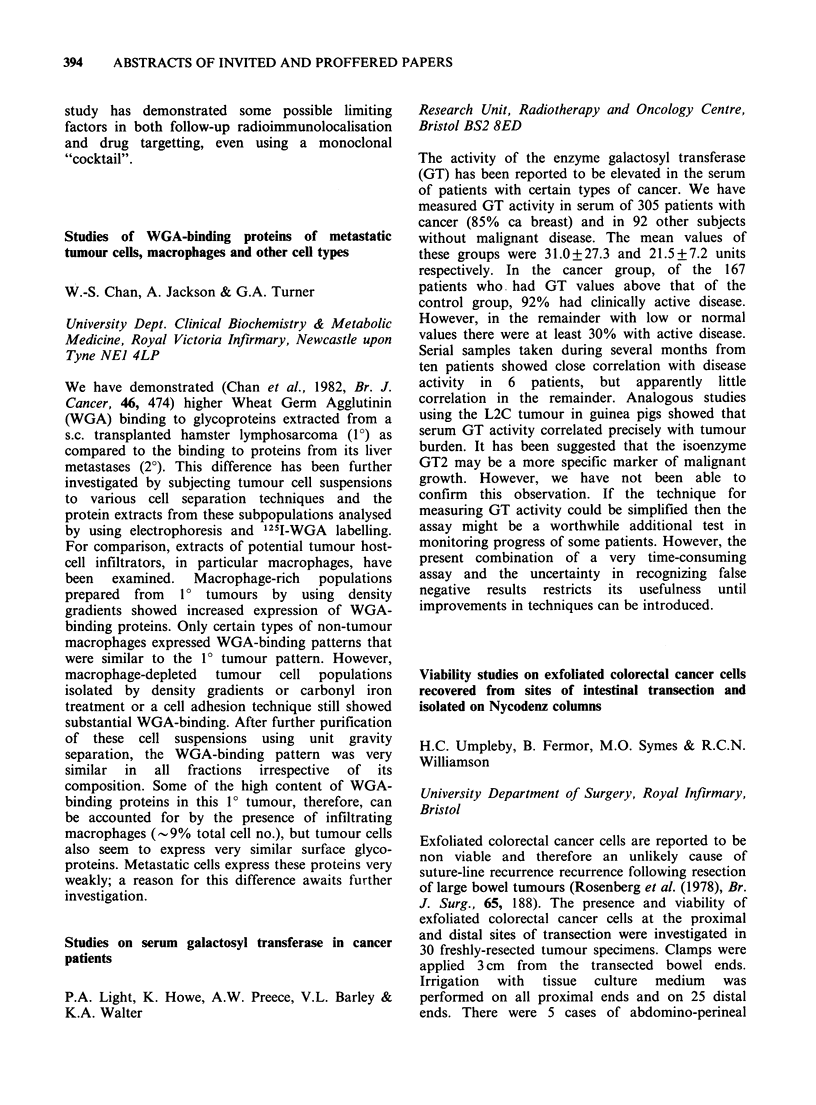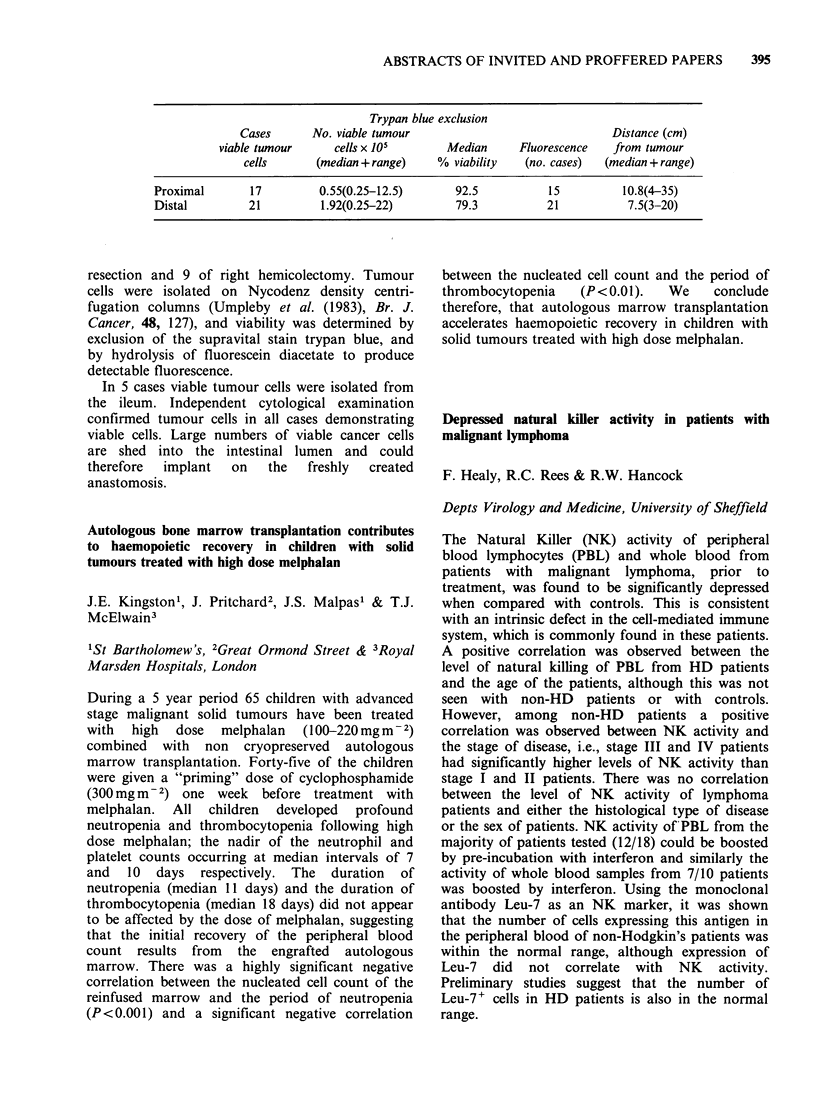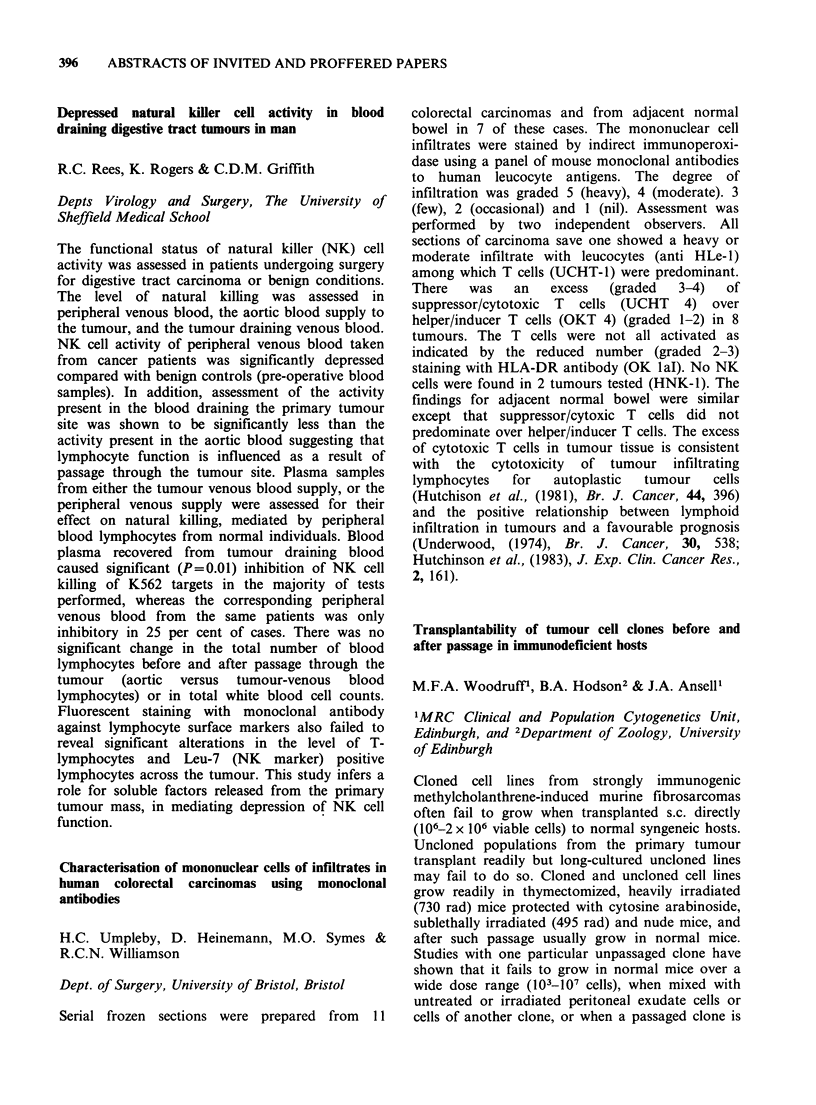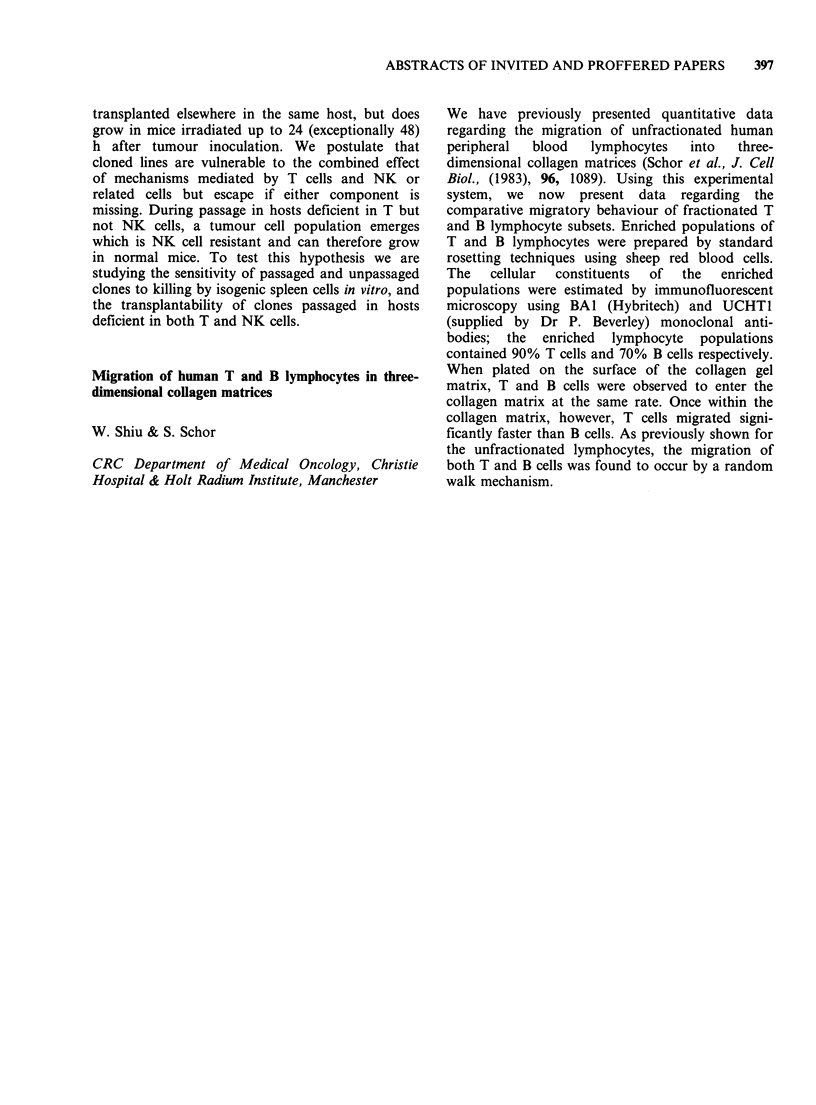# Joint meeting of the British Association for Cancer Research & the Imperial Cancer Research Fund (incorporating a symposium on "New approaches to endocrine-related cancer" and the fourth Gordon Hamilton-Fairley Lectures). November 24-25 1983.

**DOI:** 10.1038/bjc.1984.61

**Published:** 1984-03

**Authors:** 


*Br. J. Cancer* (1984), **49**, 379–397

### Joint meeting of the British Association for Cancer Research* & the Imperial Cancer Research Fund

### (Incorporating a Symposium on "New Approaches to Endocrine-Related Cancer" and the fourth Gordon Hamilton-Fairley Lecture). November 24–25, 1983

*Held at The Royal College of Physicians, 11 St Andrew's Place, Regent's Park, London, NW1. (By kind permission of the Treasurer).*

### Abstracts of Invited and Proffered Papers

The **Fourth Gordon Hamilton-Fairley Memorial Lecture**, delivered by **Dr S. Brenner, MRC Laboratory of Molecular Biology, Cambridge**, was entitled **Genes, Growth and Differentiation.**

### Endocrinology of prostatic cancer

K. Griffiths

*Tenovus Institute for Cancer Research, Heath Park, Cardiff CF4 4XX*

Despite intense investigation, the roles of the various hormones that influence the prostate gland in the aetiology of prostatic cancer remain to be elucidated. Some evidence has emerged from the work of the British Prostate Study Group to suggest that hormone analysis will allow the identification of these patients presenting with prostatic cancer who are at greater risk of rapid disease progression and more effective and aggressive treatment should probably be considered for these patients. The monitoring of hormone changes in the plasma of patients being treated is also seen to be an advantage in determining the course of therapy and a recent assessment of the use of the new LH-RH analogue (ICI 118630) for prostatic cancer therapy appears very encouraging. The value of specific antisera to proteins, isolated for various types of prostatic tissue, in drug-targetting studies, is being assessed in a number of centres and may again offer a new approach to treatment.

### The action of oestrogens and antioestrogens on cultured breast tumour cells

H. Rochefort, F. Capony, D. Chalbos, M. Garcia, F. Veith, F. Vignon & B. Westley

*Unité d'Endocrinologie Cellulaire et Moléculaire (U 148), INSERM, 60 Rue de Navacelles, 3410 Montpellier, France.*

Oestrogens and antioestrogens regulate the production of specific proteins by, and the growth of, breast cancer cells. The mechanisms involved have been studied in culture using oestrogen receptor (RE) positive cell lines derived from metastatic breast cancers. In MCF7 cells oestradiol increases progesterone receptor levels and the production of a 52,000 dalton (52 K) glycoprotein which is released into the culture medium. Oestrogens also transform the cell ultrastructure: the number and length of microvillae and the production of secretory granules are increased, while attachment of cells to plastic is decreased. Antioestrogens, Tamoxifen and Progestins, do not themselves induce the 52 K protein, but inhibit its induction by oestrogens. Using specific antibodies, the presence of this protein has been demonstrated in several pleural metastases of breast cancer and in primary breast cancer, but not in the normal mammary cell line HBL100 or in adenofibroma. Since it is secreted or shed into the culture medium, the 52 K protein is a potential circulating marker of oestrogen dependency in breast cancer. Oestrogen increases the growth of RE positive cells *in vitro* if the culture medium is sufficiently depleted of endogenous oestrogens and other mitogens. This direct effect of oestrogens in cell culture does not exclude the existence of additional indirect effects *in vivo*, mediated by growth factors produced by other tissues (oestromedins). However, it does allow the mechanism by which oestrogens stimulate the proliferation of RE positive cell lines to be studied *in vitro*. Several indirect lines of evidence suggested that oestrogens might stimulate the growth of breast cancer cells by inducing them to produce growth factors which would then stimulate their proliferation (Autocrine mechanism). We have found that non dialysable, protease sensitive, oestrogen induced factors from conditioned media

*Enquiries to the BACR Secretariat, c/o Institute of Biology, 20 Queensberry Place, London SW7 2DZ.

promote the growth of $E_2$ deprived MCF7 cells and of RE negative cells. These factors are retained on Con A sepharose. The recent preparation of monoclonal antibodies to the 52K protein or other oestrogen regulated glycoproteins released by MCF7 cells are responsible for this mitogenic activity. Antioestrogens appear to decrease the production of these factors in the medium; their action being mediated either by the oestrogen receptor (Tamoxifen) or by the progesterone receptor (Progestins).

We conclude that oestrogen responsive human mammary cell lines provide simple model systems which can faithfully reproduce the *in vivo* effect of oestrogens and antioestrogens in patients. They are therefore useful for understanding the mechanisms of hormone regulation of breast cancer and for improving its medical management.

**Molecular biology of calcitonin and katacalcin**

I. MacIntyre

*Endocrine Unit, Department of Chemical Pathology, Royal Postgraduate Medical School, Ducane Road, London, W12 0HS*

Calcitonin is synthesized by the C-cells as a large precursor (21 K). The hormone is flanked on both sides by previously unrecognised peptides. The C-terminal flanking peptide, katacalcin, is secreted together with calcitonin. The plasma concentrations of these two hormones are approximately equimolar: as expected the plasma levels of katacalcin vary with calcitonin.

Further, it has recently been shown in the rat that a different peptide is also encoded by the calcitonin gene. This 37 aminoacid peptide (CGRP) is found in the nervous system, where it may act as a neuro-modulator. Differential tissue-specific mRNA processing is apparently the explanation for the different tissue distribution reported in the rat for calcitonin and CGRP. These findings may have important physiological consequences but it remains to be determined whether a similar peptide is present in human tissues.

**Alimentary tract tumours secreting regulatory peptides**

S.R. Bloom

*Royal Postgraduate Medical School, Hammersmith Hospital, Ducane Road, London W12 0HS*

**Use of gene transfer to analyse gene expression and hormone control**

M.G. Parker

*Imperial Cancer Research Fund, P.O. Box 123, Lincoln's Inn Fields, London WC2A 3PX*

The ability to introduce DNA into eucaryotic cells has provided a means for analysing the expression of cloned genes. By manipulating the genes *in vitro*, using recombinant DNA techniques, it is possible to identify regulatory DNA sequences which are important for individual steps in gene expression. By means of this approach we are analysing the mechanism whereby testosterone regulates the expression of genes encoding prostatic steroid binding protein. The genes have been introduced into an androgen-responsive mouse cell line where their expression is accurate and, in certain cases, is stimulated up to 5-fold by testosterone. To delineate the site of action of the hormone we have constructed chimaeric genes consisting of putative C3 gene promotors and regulatory DNA sequences together with a marker gene. Expression of the marker gene was stimulated by less than two-fold by testosterone. These data suggest that in mouse cells, testosterone does not appear to modulate C3 gene expression by stimulating gene transcription and in certain clones may be acting post-transcriptionally.

In contrast, glucocorticoids stimulate the transcription of mouse mammary tumour virus by interacting with DNA adjacent to the viral promoter, even in heterologous cells. Similarly, progesterone and dexamethasone interact with a region of DNA in the egg white genes which is adjacent to the promoter but, in these cases, transcription was stimulated by steroid only in oviduct cells. Therefore, it is likely that tissue specific factors, in addition to steroid-receptor complexes, are required for hormones to stimulate the transcription of certain genes.

**New approaches to breast cancer – The potential of monoclonal antibodies**

R.C. Coombes, R. Rainsbury, W.H. Redding & A.M. Neville

*The Ludwig Institute for Cancer Research (London Branch), The Royal Marsden Hospital, Sutton, Surrey, SM2 5PX*

Monoclonal antibodies have various potential applications to diagnosis and therapy of human breast cancer.

We have investigated their use in (a) detecting micrometastases and (b) radio-localisation. In terms of the former application we are currently substituting a monoclonal antibody against milk fat globule membrane (LICR-LON-M8) for a polyclonal antiserum and it appears to have an equal ability to detect micrometastases in bone marrow.

Currently using the polyclonal antiserum, we can detect micrometastases in 28% of patients with primary breast cancer. Using the same monoclonal we have labelled it with $^{111}$Indium-diethylene-triaminepentaaceticacid complex and this has been shown to localise in bone metastases in nearly all patients with overt skeletal disease both by auto-radiography and external scanning.

A final application that we have investigated is the use of antibodies in killing breast cancer cells in bone marrow. If we could achieve this we could rescue patients with "cleaned-up" bone marrow after high-dose therapy. We have evaluated one such monoclonal (LICR-LON-Fib75) which is cytotoxic for breast cancer cells with rabbit complement. Since its effects in the colony-forming-capacity of the bone marrow are variable we are also exploring the use of toxin-linked LICR-LON-Fib75.

None of these monoclonals are breast cancer-specific and a major component of the work of this group is in trying to raise a breast-specific monoclonal reagent.

### The endorphins

L.H. Rees

*Department of Chemical Endocrinology, St Bartholomew's Hospital, London, EC1A 7BE*

Endorphins is a generic name encompassing several families of endogeneous opioid peptides, all with differing affinities for different classes of opiate receptors. Despite technical difficulties surrounding their measurement, a mass of data has accumulated implicating them in the pathogenesis of diverse conditions such as exercise-induced amenorrhoea, septic shock, chlorpropamide-alcohol flushing and alcoholism in humans as well as neurooncogenic events in experimental animals. Their secretion by phaeochromocytomas and ectopic ACTH-secreting tumours raised questions concerning their role in non-metastatic manifestations of malignant disease. Furthermore, fascinating data now exist implicating their differential release following electro-acupuncture (EAP) given for pain relief of heroin withdrawal symptoms suggesting that the electrical frequency of the EAP determines which opioid peptide is released. Naloxone, the opioid antagonist which preferentially blocks $\mu$ receptors has been extensively used in studies of the role of endogenous opioids but until good $\lambda$ and $\delta$ receptor antagonists become available the consequences of their effective antagonism will not be known. In future availability of more specific agonists and antagonists will allow manipulation of different receptor populations. Furthermore, the search for enkephalin analogues with therapeutic efficacy but without addictive properties continues unbated. These areas and many others will require further exploration before the complexity of interactions of endogenous opioids is understood. We must conclude that we are still on the threshold of understanding their physiological roles and involvement in disease states.

### Abstracts of members' proffered papers

### Flow cytometric and physical studies of glucocorticoid-induced cell death

N.O. MacKinnon[1], D.M. Lamb[1], J.E.D. Dyson[1], P. Quirke[2] & C.C. Bird[2]

[1]*Department of Radiotherapy, Tunbridge Building, Cookridge Hospital,* [2]*Department of Pathology, Leeds LS16 6QB*

The phenomenon of "programmed" cell death or apoptosis (shrinkage necrosis) has been suggested as an important factor in explaining the observed slow growth rates of tumours as compared to rates calculated on content of cycling cells (Wyllie *et al.*, 1980, *Int. Rev. Cytol.*, **68**, 251). Certain human lymphoid cell lines are thus in use as possible models for the study of apoptosis *in vitro*. Preliminary studies employing physical techniques with the CCRF-CEM and CCRF-CEM-C7 cell lines have shown changes ocurring during methyl-prednisolone-induced cell death which are consistent with observed changes in morphology during apoptosis *in vitro* and *in vivo*. Increasing condensation of cellular cytoplasm and decondensation of nuclear chromatin (assessed using fluorescence polarization and time dependent changes in staining of cellular DNA respectively) have been observed as cell death progresses. Concurrent flow cytometric studies have shown that following these changes the cells become permeable to vital dyes and then form a well defined subpopulation on the green *vs* red fluorescence scattergram. A similarly defined subpopulation is also observed when cell suspensions from whole tumours are analysed. This suggests that the latter are also apoptotic cells, which would thus allow the content of such cells in a given tumour biopsy to be estimated. Purified fractions of these subpopulations obtained by flow cytometric cell sorting are being examined by electron microscopy in order that their morphology may be compared.

**Tritiated thymidine labelling index in human prostatic cancer**

C.E. Bumford, M.E. Lambert & N.J. Blacklock

*Department of Urology, University Hospital of South Manchester, Nell Lane, Manchester M20 8LR*

Tritiated Thymidine Labelling Index (LI) is a crude but simple *in vitro* index of the proliferative activity within a tumour and thus has potential as a prognostic indicator. LIs have been determined by the method of Meyer & Bauer (1975)* on 22 specimens of human prostatic carcinoma (median LI 0.28%, range 0.0–2.77%), 37 specimens of human benign prostatic hyperplasia (median LI 0.12, range 0.0–0.65%) and 6 specimens of normal human prostate (median LI 0.04, range 0.0–0.23%).

Prostatic carcinomas of patients presenting with metastases had a significantly higher LI than those of patients without detectable metastases ($P = 0.04$ by the Kruskal-Wallis non-parametric ANOVAR); these former patients have an appreciably worse prognosis.

Although the follow-up on these patients (mean 16 months) is not adequate to assess the independent prognostic value of LI, those patients with the highest LIs in this series have rapidly advancing disease.

**Non-protein-bound estradiol, SHBG, breast cancer and breast cancer risk**

P.F. Bruning, J.M.G. Bonfrèr, D. Linders, J. van Loon & A.A.M. Hart

*The Netherlands Cancer Institute, Plesmanlaan 121, 1066 CX Amsterdam, The Netherlands*

*Meyer and Bauer (1975) etc.

It has been shown that, despite apparently normal plasma concentration of estradiol ($E_2$), the percentage of non-protein-bound $E_2$, which is supposed to be biologically active, is abnormally high in breast cancer patients. We have developed a similar assay of non-protein-bound $E_2$. Heparinized plasma was obtained from (a) 26 women at risk for breast cancer (mother and at least one sister having breast cancer); (b) 11 normal women matched for age, parity, Quetelet index and socioeconomic factors; (c) 10 women with histologically proven benign breast disease; (d) 17 women curatively treated for $T_1N_0M_0$ breast cancer at least 6 months ago. All women were premenopausal. We have collected pooled plasma in the luteal phase. The mean values $\pm$ s.d. of non-bound $E_2$ were (a) $1.78 \pm 0.28$; (b) $1.70 \pm 0.54$; (c) $1.84 \pm 0.34$ and (d) $1.86 \pm 0.40\%$ of the total $E_2$ concentrations. Differences were statistically not significant. An inverse correlation between SHBG and non-bound $E_2$ was found ($P < 0.0001$). Analysis of co-variance demonstrated that the regression lines for log SHBG vs. log non-bound $E_2$ were *identical* for the 4 groups. In 40 serum bank samples from pre- and post-menopausal patients having breast cancer the mean non-bound $E_2$ was $1.59 \pm 0.41\%$ of the total $E_2$. Log SHBG and log non-bound $E_2$ showed again very good correlation; the regression line ran parallel with those of groups A–D though significantly lower. Our preliminary results confirm a very strong inverse correlation between non-bound $E_2$ and SHBG. There is no difference in non-bound $E_2$ between premenopausal women at risk for breast cancer, normal matched controls, women with benign breast disease and patients cured for early breast cancer or having breast cancer.

**Peroxidase activity, a possible prognostic marker in breast carcinoma**

M.J. Duffy, M. O'Connell, G.J. Duffy, B. Cantwell, J. Fennelly, L. O'Siorain & R. Conroy

*Depts. of Nuclear Medicine & Oncology, St Vincent's Hosp. Dublin 4*

Peroxidase activity is massively induced in the immature rat uterus following estradiol administration. If a similar induction occurred in breast carcinomas peroxidase could be a marker for a functional estradiol receptor (ER) and thus be of great value in predicting hormone-dependent tumours. Although peroxidase activity has been reported to be higher in some hormone-dependent animal breast tumours than in hormoneindependent tumours, no correlation has been found between ER and peroxidase activity in human breast carcinomas. Thus peroxidase is unlikely to be a marker for predicting hormone-dependent human breast tumours.

On the other hand peroxidase activity may be a prognostic marker in breast cancer. In this investigation, patients whose tumours possessed peroxidase activity had both shorter disease free interval and shorter survival than patients with peroxidase-negative tumours. These differences were statistically significant; for disease free interval $P=0.0474$; for survival $P=0.0165$. Patients with ER-positive tumours also had a longer disease-free interval and longer survival than ER-negative tumours. However since ER were mostly confined to low grade and low stage tumours, this protein may not be a totally independent prognostic marker. In contrast, peroxidase activity showed no relationship to tumour stage or grade. It may however be a measure of lymphocyte infiltration of the tumour. (Duffy *et al.*, 1982 *Eur. J. Cancer*, **18**, 453). Conclusion: Although peroxidase activity is unlikely to be a marker for hormone-dependent breast tumours, its presence in these tumours is a bad prognostic feature and its absence suggests a good prognosis.

### Increased activity of dihydropteridine reductase in human breast tumours

J.A. Blair, P.A. Barford, C. Eggar & G.D. Oates

*Department of Chemistry, University of Aston, Birmingham, B4 7ET and The General Hospital, Birmingham, B4 6NH*

The enzyme dihydropteridine reductase (DHPR) is widely distributed in mammalian tissues. Its known functions are the reduction of quinonoid dihydrobiopterin to tetrahydrobiopterin and quinonoid dihydrofolate to tetrahydrofolate.

Measurement of DHPR activity in human breast tumours and matched adjacent apparently non-malignant tissue obtained at operation shows that DHPR activity is very significantly higher in the tumour than in the control tissue. Similar measurements with human gut cancers show no difference between tumour and control tissue.

In the rat large doses of oestrogens increase tissue DHPR activity. In man measure erythrocyte DHPR activity increases with increased exposure to oestrogens. It has been previously reported that in human breast tumours DHPR activity correlates well with oestrogen receptor density.

It therefore appears likely that the increased DHPR activity in human breast tumours is due to the increased uptake by the tumour tissue of oestrogen. Measurement of DHPR activity in breast tumours and its comparison with normal adjacent tissue may be a valuable procedure in the prognosis and treatment of breast cancer.

### Studies on the differentiation of human astroglioma cells

M. Weir, A. Hunt, A.J. Patel & D.G.T. Thomas

*Department of Neurological Surgery and MRC Developmental Neurobiology Unit, Institute of Neurology, Queen Square, London WC1N 3BG*

The life expectancy of a patient with a low grade astrocytoma is much greater than one with a high-grade tumour. Since low grade astrocytoma contain relatively more differentiated cells, the understanding of differentiation in astroglial cells would be clinically useful. Recently, we have shown that the developmental increases in the astrocyte enriched proteins, glutamine synthetase (GS) and glial fibrillary acidic (GFA) protein are related to maturation rather than proliferation of astrocytes. These proteins were used to study the effect of various metabolic factors on the differentiation of glioma and normal astrocyte cells in culture. In human glioma cell line U251MG ethanol treatment was found to decrease GS and increase GFA protein concentrations in a dose dependent manner ($IC_{50}$, 0.05–0.1%). In contrast, alcohol had no effect on either the morphology (at the light microscope level), or cell numbers, or total protein content of these cells. The effect of alcohol could be prevented by concomitant addition of dexamethasone (DEX). Similar effects of alcohol was observed in 10 primary cultures derived from various grades of astrocytoma. The effect on astrocyte-enriched proteins may be specific to transformed cells as alcohol (up to 1%) has little appreciable effect on normal mammalian astrocytes. Other factors studied were addition of DEX and removal of glutamine from culture medium; both resulted in a major increase of GS protein, though tumours varied in their sensitivity. These effects do not seem to be specific to the transformed cells, since both the removal of glutamine and addition of DEX resulted in a marked increase in GS in normal astrocyte cultures. Furthermore both these effects were additive in all types of astroglial cells. These results suggest involvement of more than one mechanism in the regulation of astrocyte enriched proteins in both astroglioma and normal astrologlial cells.

### Suppression of growth of a human tumour xenograft by a vindesine monoclonal antibody conjugate

M.V. Pimm[1], G.F. Rowland[2], R.G. Simmonds[2], H. Marsden[2], M.J. Embleton[1], E. Jacobs[1] & R.W. Baldwin[1]

[1]*Cancer Research Campaign Laboratories, University of Nottingham,* [2]*Lilly Research Centre Ltd., Windlesham, Surrey*

The production of anti-tumour monoclonal antibodies has prompted the development of conjugates with anti-tumour agents for specific targeting to tumour sites. Monoclonal antibody 791T/36 (mouse IgG2b), against the human osteogenic sarcoma cell line 791T, was radiolabelled with $^{125}$I, and localized in xenografts of 791T in immunodeprived mice. Kinetic studies showed that the maximum degree of localization was achieved 3 to 4 days after antibody administration. The maximum tumour level of antibody achieved was $80\,\mu g\,g^{-1}$ of tissue following injection of 100–$200\,mg\,kg^{-1}$ body weight of 791T/36 antibody.

The vinca alkaloid Vindesine (VDS) was conjugated covalently to 791T/36 antibody (3:1 to 6:1 molar ratios). These conjugates were cytotoxic *in vitro* specifically for target cells expressing the 791T/36 defined antigen and $^{125}$I labelled conjugates localized *in vivo* in 791T xenografts. The therapeutic effects of conjugate was tested against 791T xenografts. Mice were injected at 3 to 4 day intervals with up to $180\,mg\,kg^{-1}$ 791T/36–$5\,mg\,kg^{-1}$ VDS/injection (total doses up to $1.6\,g\,kg^{-1}$ 791T/36–$45\,mg\,kg^{-1}$ VDS) and this significantly retarded tumour growth with no toxicity to the mice. Free antibody had no influence on tumour growth. Free VDS was significantly tumour suppressive at doses equivalent to those in the conjugate, but was also markedly toxic to the mice since the doses used exceeded the established acute LD50 of the drug ($6.3\,mg\,kg^{-1}$, Todd *et al.*, 1976 *J. Toxicol. Environ. Health*, **1**, 843).

These studies suggest that drug-antibody conjugates could have considerable potential for selective anti-tumour therapy.

### Human tumour cells "escaping" a cytotoxic monoclonal antibody-methotrexate conjugate do not constitute permanently resistant clones

M.J. Embleton, E. Jacobs, M.C. Garnett & R.W. Baldwin

*Cancer Research Campaign Laboratories, University of Nottingham, Nottingham NG7 2RD*

In evaluating monoclonal antibody-drug conjugates as anti-tumour agents, one potential problem is the emergence of resistant tumour cell clones. In addition to selection for drug-resistance it is possible that clones of diminished antigenicity could arise as a result of modulation or selection. We have measured both parameters in "resistant" clones of a human osteogenic sarcoma line (791T) isolated after treatment with a monoclonal antibody-methotrexate (MoAb-MTX) conjugate.

Parental cells exposed at low density *in vitro* to MoAb-MTX containing the equivalent of 50 or $100\,ng\,ml^{-1}$ methotrexate (MTX) showed plating efficiencies of 0.28% and 0.08% of control levels, respectively. Of 15 colonies isolated at these doses and subsequently propagated, 11 failed to undergo more than a few cell divisions and only 4 developed into cloned lines. The "resistant" clones were assayed for antigenicity by flow cytometry using fluorescein isothiocyanate-conjugated monoclonal antibody, and for drug-resistance by assessing their plating efficiency in free MTX. Two clones showed antibody binding within the normal range for the parental 791T line, and two were more antigenic. Three of the clones were as susceptible to MTX toxicity as 791T cells ($IC_{50}$ 8 to $10\,ng\,ml^{-1}$) and the fourth was only slightly less sensitive ($IC_{50}$ $17\,ng\,ml^{-1}$). These findings suggested that the clones would not resist a further exposure to the MoAb-MTX conjugate, and this was confirmed by showing that the $IC_{50}$ of conjugate for both parental cells and clones was in the range 1.5 to $3\,ng\,ml^{-1}$ (in terms of MTX).

It is concluded that cells emerging after exposure to a cytotoxic conjugate are not necessarily resistant, but may either be incapable of unlimited growth or sensitive to further attack by the same conjugate.

### Comparison of *in vitro* chemosensitivities of continuous cell lines derived from transitional cell cancers of the human bladder

P.J. Hepburn & J.R.W. Masters

*Dept. Histopathology, Institute of Urology, St Paul's Hospital, London WC2H 9AE*

The aim of this study was to measure the *in vitro* sensitivities to chemotherapeutic drugs of continuous cell lines derived from human tumours of one histological type. Eight lines derived from transitional cell cancers of the human bladder, MGH-U1, MGH-U2, T24, RT112, TCCSUP, 253J, HT1376 and RT4, were exposed to a cell cycle phase-specific drug, methotrexate and a cell cycle specific drug, adriamycin. The *in vitro* sensitivities following exposure for 24 h to a range of drug concentrations were measured using a clonogenic assay on plastic. We have shown that three of these lines, MGH-U1, MGH-U2 and T24 are cross-contaminated (O'Toole *et al.*, 1983, *Nature,* **301,** 429). The *in vitro* sensitivities of these three lines were similar, but the remaining five lines showed a wide range of response. The proportion of clonogenic cells surviving exposure to $100\,ng\,ml^{-1}$ methotrexate ranged from 1.5% for MGH-U1 to 89.5% for HT1376, and to $30\,ng\,ml^{-1}$ adriamycin from 0.7% for MGH-U1 to 42% for HT1376. The sensitivities of the cell lines showed the same rank order for each drug. Cytotoxicity was also related to growth rate, in that the higher the population doubling time and colony forming efficiency the more sensitive were the cells to each drug. In conclusion it has been shown that continuous cell lines derived from one histological type of tumour show a wide range of drug sensitivities. Therefore, data derived from one cell line may not accurately reflect the drug sensitivities of tumours of that histological type.

### Are the drugs that have been stored in solution still active when added to an *in vitro* chemosensitivity assay? A review with special reference to nitrosoureas and nitrogen mustards

A.G. Bosanquet

*Department of Clinical Investigation, Royal United Hospital, Combe Park, Bath BA1 3NG*

Many drugs are required in solution at short notice for an *in vitro* chemosensitivity assay. They are often made up in phosphate buffered saline (PBS) or saline and stored frozen until required. However, (a) it has been suggested that solutions of BCNU are unstable under these conditions, (b) some drugs have very short half lives in solution, and (c) I have found that aqueous solutions of 2,5-diaziridinyl-3,6-bis(2-hydroxyethyl-amino)-1,4-benzoquinone (BZQ) irreversably precipitate on being frozen. These instances suggest that care ought to be taken in the preparation and storage of drug solutions for *in vitro* use. Some drugs are very stable in solution (prednisolone, cytosine arabinoside) so that they can be stored at 4°C for some months with no detrimental effects; solutions of others need to be stored frozen, but 1–2 h at room temperature will not affect them significantly (vincristine, vinblastine, vindesine bleomycin, adriamycin, actinomycin D, methotrexate) whilst the nitrogen mustards and nitrosoureas must be treated with care. It is suggested that nitrosoureas be stored in ethanol solution at −40°C or lower and diluted in saline pH 5 on the day of the assay to be most stable; BCNU is slightly less stable than CCNU and MeCCNU. Nitrogen mustards can be frozen in solution, but not left at room temperature for any length of time. The values of $t_{0.95}$ (5% degraded) in PBS (pH 7) at 25°C for chlorambucil, melphalan, nitrogen mustard and 4-hydroperoxycyclophosphamide are about 15, 45, 45 and $\geqslant 1$ h respectively.

### A mechanism for folate catabolism

J.A. Blair, P.A. Barford, A. Sahota, C. Morar, M. Surdhar & D. Al-Haddad

*Department of Chemistry, University of Aston, Birmingham B4 7ET*

It is now well established that folate catabolism occurs in man, rat, guinea pig and hamster giving p-acetamidobenzoate and p-acetamidobenzoyl-L-glutamate as metabolites derived from the aminobenzoyl-L-glutamate moiety and urea, $CO_2$ and unidentified fragments from the pterin portion. In man and the rat this catabolism is decreased in the presence of a tumour and in the rat is increased by the administration of methotrexate, an inhibitor of the reductases which maintain the tetrahydrofolate pool. It is therefore important to establish the mechanism of this catabolism.

Tetrahydrofolate is oxidised by dioxygen in aqueous solution at neutral pH giving pterin and p-aminobenzoyl-L-glutamate as scission products by a free radical chain mechanism with superoxide anion as chain carrier.

Tetrahydrofolate and tetrahydrobiopterin (a model for tetrahydrofolate) are oxidised at an increased rate in the presence of xanthine plus xanthine oxidase and this oxidation is significantly reduced by superoxide dismutase. Thus tetrahydrofolate can be oxidised to scission products by superoxide anion formed from an enzyme reaction.

In the hamster folate catabolism is significantly increased after administration of large amounts of allopurinol, xanthopterin and dihydroorotate, compounds whose *in vivo* metabolism may increase *in vivo* superoxide anion formation. Similar results are obtained in the rat after doses of allopurinol.

Folate catabolism is therefore due to oxidation of tetrahydrofolate by superoxide anion formed by enzymic, phagocytic and chemical oxidation.

### Plasma and tumour pharmacokinetics of benznidazole in man

J.T. Roberts, N.M. Bleehen, P. Workman & M.I. Walton

*University Dept. and MRC Unit of Clinical Oncology and Radiotherapeutics, Addenbrooke's Hospital, Hills Road, Cambridge CB2 2QQ*

Benznidazole has been reported to enhance the response of mouse tumours to CCNU at plasma and tumour concentrations which should be readily attainable in man (Twentyman & Workman, (1983) *Br. J. Cancer*, **48**, 17). This enhancement is considered greater than that seen in normal tissues, resulting in a net therapeutic gain. As part of a phase I assessment of this combination we have determined the plasma, urine and tumour pharmacokinetics of benznidazole by reverse phase HPLC. Twenty-six patients have received benznidazole in combination with CCNU, the benznidazole dose being increased in successive groups of patients from $4\,mg\,kg^{-1}$ to $30\,mg\,kg^{-1}$. Both drugs were given orally, benznidazole preceding CCNU by 3 h. No evidence of saturation kinetics has been seen at doses currently used. The mean plasma half life ($t\frac{1}{2}$) was $12.8 \pm 0.5$ h (s.e., $n=25$). Plasma peak concentration and AUC were linearly related to dose over the whole range. As an example the average peak plasma concentration at $8\,mg\,kg^{-1}$ was $13.6 \pm 1\,mg\,kl^{-1}$ ($n=7$). The median peak time was 4 h. Approx. 60% of the drug was protein bound. Approx. 6% of the dose was excreted unchanged in the urine, the rest being unaccounted for. Benznidazole concentrations have been measured, at varying times, after oral administration of the drug, in biopsies from 11 patienrs with brain tumours and 6 patients with tumours in other sites. A plateau of maximum concentration is seen over 2–6 h with mean tumour plasma ratios of 88% for gliomas and 72% for non-brain tumours. Absolute tumour concentrations of $8\text{–}9\,mg\,ml^{-1}$ were readily achieved with doses of $8\,mg\,kg^{-1}$. We have shown that it is possible to achieve in man plasma and tumour levels of benznidazole which, in the mouse model, produce effective enhancement of CCNU response.

### An assessment of staging procedures in patients with small cell carcinoma of the bronchus (SCCB)

S.P. Lockhart, M.A. Cornbleet, M.V. Merrick[1], S.G. Allan, R.C.F. Leonard & J.F. Smyth

*Dept. of Clinical Oncology, [1]Dept. of Nuclear Medicine, Western General Hospital, Edinburgh EH4 2XU*

Seventy patients with histologically proven SCCB were investigated to determine the value of limited staging (LS), clinical with haematological and biochemical evaluation against full staging by including bone scanning (BS), liver ultrasound (LUS) and bone marrow (BM) examination. BS was performed on 56 patients and positive in 16, being the only positive procedure in 9 of those patients fully staged. Alkaline phosphatase (AP) and plasma calcium were not useful indicators of bone disease. The result of BS could not be predicted reliably from limited staging. LUS was performed on 65 patients, positive in 12, being the only positive staging test in just one patient. AP and AST were not useful in predicting LUS results but a normal LDH was highly correlated with normal LUS. BM was performed on 47 patients and abnormal in 4. This was not predicted by blood count but was associated with elevated LDH. Overall, 44 patients had all staging procedures. Compared with LS, 15 were downstaged and 5 upstaged as a result of further procedures. After full staging, 16 had extensive and 28 limited disease. Elevated LDH was present in 9/16 patients with metastatic disease but was also present in 8/28 patients with limited disease. Normal LDH correctly predicted a normal LUS; unexpectedly, elevated LDH was associated with abnormal BM. Routine use of all investigations is expensive, time-consuming and unnecessary for all patients. This study indicates that using limited staging plus BS, with BM and LUS only for those patients with elevated LDH, only 2/44 patients would have been incorrectly staged.

### Methods of evaluating quality of life in cancer patients

P. Selby[1], J.-A. Chapman[1] & N.F. Boyd[2]

*[1]Department of Medicine, Institute of Cancer Research, The Royal Marsden Hospital, Sutton, Surrey, [2]The Princess Margaret Hospital, Toronto, Canada*

Although the need to directly measure quality of survival in the investigation of cancer treatment is widely recognised, satisfactory methods have not been established and their evaluation remains complex and indirect. We have studied the use of a self assessment measurement method in breast cancer patients. Sixteen items describing general health features were drawn from the Sickness

Impact Profile, a lengthy established method for measuring functional status, 15 items describing features of breast cancer were drawn from patients opinions and clinical experience. Each item was assessed by a linear analogue scale. The method was shown to be reliable with test-retest correlations of >0.7 for 23 items (Selby *et al.*, 1982, *Proc. ASCO*, p. 45) and internally consistant.

Validity of items cannot be established directly since no standards exist. Estimates of validity were made indirectly by comparisons with other methods, by comparisons between items using factor analysis and by comparisons with physician scores in a total of 177 breast cancer patients.

Patients scores were generally highly correlated to physician assessments with coefficients >0.6 in 25/31 items although the variance of patients scores was greater than that of physicians. Scores were highly correlated to scores in equivalent categories of the Sickness Impact Profile with 6/7 coefficients >0.6 for items which were directly comparable. The factorial composition of the items' scores revealed 5 closely inter-correlated groups of items compatible with clinical experience.

**The assessment of quality of life in cancer patients; a fresh approach**

S. Bindemann, R.A.V. Milsted, S.B. Kaye & K.C. Calman

*Department of Clinical Oncology, University of Glasgow, Glasgow 12*

Quality of life is highly relevant to management of the Ca patient. However, difficulties in its assessment are well known. This Department has attempted an improved method by means of a self-report questionnaire. A sample of Ca patients (*n*100) and of control subjects (*n*80) were asked to describe "the kind of person I am" by selecting items from a "pool" of bi-polar items, e.g. "happy-sad" etc. Subjects were then required to describe perception of their ideal self, i.e. "the kind of person I should like to be". Factor analysis provided a statistical method for data reduction into clusters of homogeneous items or factors. A two-factor solution was adopted, *viz.* a factor of "intrapsychic functioning" and a factor of "anticipation of the future". Taking the first factor only, as an example it is apparent that at its pole of "unsatisfactory functioning", items such as "often depressed", "seldom relaxed" etc. were the items chosen. Conversely, the "satisfactory functioning" pole was characterised by their polar alternatives, i.e. "seldom depressed" etc. Computation of factor-scores facilitated group values which indicate position on a linear scale, having a zero mean with unit s.d.'s $\pm$ on either side. Results indicate statistically significant discrepancies between Ca patients and control Ss (subdivided into non-Ca patients (*n*30) and non-patient Ss (*n*50)) in "intrapsychic functioning" scores (analysis of variance $= P < 0.05$). Differences between these discreet groups (using students' *t*) were as follows: Ca patients and non-Ca patients $P = < 0.01$; Ca patients and non-patient Ss $P = < 0.01$. Statistically significant differences also differentiated Ca patients from control Ss in the size of discrepancy between actual and ideal self-perceptions (invariably greater in the case of Ca patients). We hypothesise that refinement of the measurement of such self-perceptions by means of a self-evaluation questionnaire will greatly enhance assessment of quality of life.

**The effect of dose on the absorption of oral etoposide**

V.J. Harvey, M.L. Slevin, S.P. Joel, A. Johnston & P.F.M. Wrigley

*Imperial Cancer Research Fund Department of Medical Oncology, St Bartholomews and Hackney Hospitals and Department of Clinical Pharmacology, St Bartholomews Hospital, London EC1A 7BE*

Etoposide is a semi-synthetic podophyllotoxin derivative active in a variety of malignancies. It is frequently administered orally but bioavailability via this route is variable. The effect of dose on the absorption of etoposide is unknown and has therefore been studied in six patients with lung carcinoma, acting as their own controls. All were ambulant with normal hepatic and renal function. Patients were fasted for 12 h prior to treatment with doses of 200, 400, and 600 mg using oral capsules on 3 consecutive days. The order of treatment was randomised. The Area Under the Curve (AUC) was proportionally greatest at 200 mg. Doubling the dose from 200 mg to 400 mg increased AUC by only 44.2% and a further increase of 13.5% occurred at a dose of 600 mg. These data indicating non-linear absorption of etoposide within the range in clinical use may explain variations in the results of reported studies. They may have important implications for those chemotherapy regimens using oral etoposide.

**Etoposide containing combination chemotherapy for Hodgkin's disease**

P. Selby, B. Robinson & T.J. McElwain

*Department of Medicine, Institute of Cancer Research and The Royal Marsden Hospital, Downs Road, Sutton, Surrey*

Etoposide has been shown to be active in Hodgkin's disease (Taylor *et al.*, 1982, *Cancer Chemo. Pharmacol.*, **7**, 175). We have explored the combination of vincristine (Oncovin) 1.4 mg m$^2$ i.v. days 1 and 8, prednisolone 40 mg orally daily for 14 days, etoposide 200 mg m$^{-2}$ orally daily for 5 days and chlorambucil 6 mg m$^{-2}$ orally daily for 14 days (OPEC) in an attempt to increase the efficacy and decrease the toxicity of treatment.

Thirty-nine patients (20 clinical stage (CS) IV, 9 CS III, and 10 CS II with poor prognostic features) were treated. Thirty patients including 28 previously untreated, 1 previously treated with radiotherapy (RT) and 1 with chemotherapy (CT) 9 years earlier, received OPEC alternating with ChlVPP, an established quadruple CT regimen. Ten CS II patients also received RT. 23/70 (77%) entered complete remission (CR). Nine patients who had received previous chemotherapy were treated with OPEC alone and 8 entered CR. Updated results of remission duration will be presented. Nineteen of 39 patients reported nausea, 11/39 vomiting and 39/39 alopecia. Myelosuppression was mild, delaying treatment in only 7 patients. Etoposide can be given in full dosage in combination and the initial results indicate acceptable efficacy.

An alternative regimen with adriamycin, vincristine, prednisolone, etoposide and bleomycin (HOPE-BLEO) has been piloted in 8 relapsed and resistant patients. A safe schedule has been established and initial results support its further investigation.

### Management of Hodgkin's disease in children, with ChlVPP and involved field radiotherapy

B. Robinson, J. Kingston, T.J. McElwain, J.S. Malpas & A. Barrett

*Dept. Medical Oncology, St Bartholomews Hospital, London, Royal Marsden Hospital, Sutton, Surrey*

Eighty-two children, with Hodgkin's disease and no previous treatment were treated at the Royal Marsden (43) and Saint Bartholomew's (39) Hospitals from 1974 to 1982. All had histology review, full clinical staging, and 20 were pathologically staged. Treatment involved field radiotherapy, preceded by ChlVPP in some, for Stage IA and Stage IIA with favourable histology and small bulk. Stage IIA with more than 3 sites involved or bulk disease, and Stage III were treated with ChlVPP (6 courses) and radiotherapy of bulk disease, and Stage IV with 10 courses ChlVPP. ChlVPP is chlorambucil, 6 mg m$^{-2}$, 0, daily, procarbazine, 100 mg m$^{-2}$, 0, daily and prednisolone, 25 mg m$^{-2}$, 0, daily, for 14 days; vinblastine, 6 mg m$^{-2}$, i.v. days 1 & 8.

The 58 males and 24 females (ratio 2.4:1) had a mean age of 10.6 years, range 2.7–15.9 years. Lymphadenopathy was a presenting feature in 90%, B symptoms in 22%. Nodular sclerosis predominated in both sexes, 60% overall in contradistinction to our previous experience. Stage distribution was: I,15; II,30; III,24; IV,13. Complete remission was documented in 79 or 96%, PR in 2, PD in 1. At 5 years, survival was 93% and relapse-free survival 84%, median follow up >4.5 years. ChlVPP was non-toxic. Four died of HD, 1 of pneumocystis carinii, 1 of pneumococcal septicaemia 5 months after splenectomy. New protocols aim to minimise late effects on fertility, bone growth, induction of neoplasia and avoid splenectomy which was associated with a 2-fold greater infection risk.

### Effects of cytotoxic therapy on proximal jejunal absorptive function in man

D. Cunningham, L.M. Nelson, R.J. Morgan, P.R. Mills, M. Soukop, C.S. McArdle & R.I. Russell

*Gastroenterology Unit, Department of Medical Oncology and Surgery, Glasgow Royal Infirmary*

Cytotoxic therapy induces morphological changes in the proximal intestine, being maximal between 24 and 48 h after intravenous administration. However, there is little published information on the associated changes in function.

We have studied 6 patients receiving adjuvant chemotherapy for adenocarcinoma of breast. Using a triple lumen tube perfusion system, the absorption of water and electrolytes before, and 45–48 h after, administration of the i.v. cytotoxic agents cyclophosphamide (300 mg m$^{-2}$), methotrexate (40 mg m$^{-2}$) and 5-fluorouracil (600 mg m$^{-2}$) were measured. Median (range) water absorption dropped from 126 (40–142) to 72 (46–142) ml per h per 30 cm, but this was not statistically significant with similar results for electrolytes (Table I).

**Table I** Absorption of water and electrolytes before and after chemotherapy

| Pat. No. | *Water* $ml.h^{-1}$ $30\,cm^{-1}$ *Before* | *After* | *Sodium* $mmol.h^{-1}$ $30\,cm^{-1}$ *Before* | *After* | *Chloride* $mmol.h^{-1}$ $30\,cm^{-1}$ *Before* | *After* |
|---|---|---|---|---|---|---|
| 1 | 103 | 142 | 10.7 | 12 | 9 | 12 |
| 2 | 123 | 84 | 10 | 9 | 9 | 8 |
| 3 | 142 | 84 | 14 | 6 | 11 | 6 |
| 4 | 137 | 46 | 10 | 6 | 12 | 3 |
| 5 | 40 | 55 | 3 | 5 | 3 | 5 |
| 6 | 129 | 98 | 14 | 10 | 13 | 9 |

In this study no consistent change in absorptive function of the proximal jejunum following chemotherapy was demonstrated.

### The viability of marrow kept at 4°C

J.L. Millar, G. Joshi, R.D. Clutterbuck & I.E. Smith

*Institute of Cancer Research and Royal Marsden Hospital, Sutton, Surrey*

Very often, in autologous bone marrow transplantation, the marrow cannot be returned to the patient for some time after aspiration. This is generally because the drug(s) given to the patient have a finite clearance time and the marrow cannot be reinfused until the drug(s) have reached a non toxic level. During this time the marrow could be cryopreserved but this results in loss of stem cells, is costly and time consuming. The alternative is to refrigerate the marrow until it can be returned but clearly there is a limit to how long it will remain viable. In both human and mouse marrow it is possible to measure progenitor cells committed to the granulocyte/macrophage pathway (GM-CFC) but this in itself is no sure test of marrow viability. We performed parallel studies between refrigerated human marrow and mouse marrow in their capacity to produce GM-CFC with time at 4°C. In addition we used the mouse marrow to repopulate lethally irradiated mice. The mouse marrow inoculum was chosen to be just sufficient to keep the mice alive at the beginning of the experiment so that any decline in repopulating ability was reflected in terms of reduced animal survival. The GM-CFC numbers of both mouse and human marrow decreased linearly with time as did the repopulating capacity of the mouse marrow, all three declining to $\sim$5% of the starting values by 72 h. This suggests that GM-CFC may accurately reflect repopulating ability and that the half-life of marrow stored at 4°C is $\sim$24 h.

### Are cerebrospinal neurotransmitters related to neurotoxicity in children receiving treatment for acute lymphoblastic leukaemia?

C.R. Pinkerton[1], I. Smith[2], R.J. Leeming[3], G. Curzon[4] & G. Sarna[4]

[1]*Department of Haematology & Oncology, Hospital for Sick Children, Gt Ormond St., London,* [2]*Institute of Child Health, Guilford St., London,* [3]*Dept. Haematology, Birmingham General Hospital,* [4]*Dept. Neurochemistry, National Hosp., London*

A variety of neurological syndromes have been described in children with ALL and appear to be related to chemotherapy and/or cranial irradiation. It has been suggested that a possible contributory factor is the inhibition of central neurotransmitter synthesis by methotrexate due to its effects on reductase enzyme systems (Abelson, 1978, *Cancer Treat. Rep.*, **62**, 1999). To test this hypothesis the concentrations of plasma biopterins, CSF biopterins, homovanillic acid and 5 hydroxindolacetic acid were estimated in 77 children with ALL at various stages of treatment. Crithidia bioassay was used for total biopterin measurement and HPLC for HVA and 5 HIAA. There was a significant elevation of plasma biopterins ($P<0.001$) associated with chemotherapy which persisted during maintenance treatment. This was not, however, accompanied by a corresponding decrease in HVA and 5HIAA. Moreover the trend in treated cases was towards higher levels than anticipated when corrected for the patient's age. This has been described in the experimental animal and it seems likely that MTX independent pathways are utilized to maintain CSF neurotransmitter levels (Nichol, 1983, *Proc. Natl Acad. Sci.*, **80**, 1546). There was no evidence in patients studied sequentially that cranial irradiation altered CSF biopterins, HVA or 5HIAA. It seems unlikely therefore that the prophylactic administration of neurotransmitters such as L-dopa, carbidopa or 5HT would protect against neurotoxicity, as has been previously suggested (Cotton, 1978, *Lancet*, **ii**, 484).

### Recombinant DNA human interferon alpha 2 (IFN) in advanced breast cancer (abc)

N. Padmanabhan, F.R. Balkwill, J. Bodmer & R.D. Rubens

*ICRF Breast Cancer Unit, Guy's Hospital and ICRF Laboratories, Lincoln's Inn Fields, London*

Patients (pts) with evaluable progressive abc have been treated in a randomised phase 2 trial to receive IFN either 2 megaU $m^{-2}$ $day^{-1}$ sc 3 × wk (S1) or 50 megaU $m^{-2}$ $day^{-1}$ iv in 50 ml saline in 30 min on Days 1 to 5 Q 3 wks (S2). 11 pts have been accrued so far. Ten pts had prior chemo ± endocrine therapy (Rx) for abc, while 1 had adjuvant chemoRx only. Responses were assessed by UICC criteria. In 5 pts on S1, duration of Rx varied from 5 to 21 wks. All pts progressed (pd) on Rx. Six pts on S2 received 2 to 8 courses, 5 pd on Rx & 1 had stable disease. Toxicity included fever, chills, rigors, headache, myalgia, anorexia, nausea, vomiting, tiredness, somnolence and weakness. Bone marrow depression was seen in S1 and 2 but dose modification was necessary only in S2. All pts had transient elevation of liver enzymes. Hyperglycemia (pt not known to have diabetes prior to IFNRx), peripheral neuropathy (pt with stable diabetes but without overt neuropathy prior to IFNRx) and heart failure (pt without overt heart disease but had received adriamycin in the past) were noted in 3 different pts on S2. Pharmacokinetic studies in pts on S2 showed a sharp rise in serum IFN with a peak at 1 hr (1–4.5 KU $ml^{-1}$) followed by a rapid decline (mean 1/2 life – 2.5 h). Levels of peripheral blood lymphocyte's surface HLA antigen were studied in 7 pts on Days 0, 1, 3 and 5, using monoclonal anti HLA-ABC and $\beta 2$ microglobulin antibodies. 2/3 pts on S2 showed a marked rise in reactivity by Day 5 (1:800 to 1:6400). 1/4 pts on S1 showed a marginal increase and 3 none. In conclusion, IFN in 2 different regimens has not shown any evidence of activity against abc after prior systemic Rx. (IFN was supplied by Schering-Plough Corp.)

### Mitoxantrone, a Phase II study in advanced breast cancer

R.E. Coleman, M.N. Maisey, R.K. Knight & R.D. Rubens

*ICRF Breast Cancer Unit, Guy's Hospital, London*

Mitoxantrone is an anthracenedione with some structural similarities to Adriamycin. Thirty-four patients with advanced breast cancer, who had not received previous chemotherapy for advanced disease were treated with Mitoxantrone 14 mg $m^{-2}$ i.v. every 21 days. Before commencing treatment, and 3-monthly thereafter, measurements of ventricular ejection fraction by gated blood pool angiocardiography was performed at rest and in response to stress (cold-pressor and isometric hand-grip). Eleven of 33 evaluable patients (33%) achieved a partial response. There were no complete responders. The dose limiting toxicity was marrow suppression. Dose reduction was necessary in 106/220 courses (48%). Septicaemia occurred in 3 patients and was fatal in 2. Nausea and vomiting was mild and transient (WHO grades 1 and 2) in all but one patient. Two patients developed marked allopecia. ⩾15% deterioration in ejection fraction at rest and/or following stress was seen in 10 patients. Two patients developed reversible cardiac failure. Mitoxantrone is an active, well tolerated agent in the treatment of advanced breast cancer. Cardiotoxicity does occur but the precise nature and incidence requires further evaluation.

### The treatment of advanced breast cancer with an analogue of gonadtrophin releasing hormone

J.H. Waxman, M.L. Slevin, R.C. Coombes[1], S. Harland[1], J.S. Malpas, P.M.F. Wrigley & T.A. Lister

*St Bartholomew's and Royal Marsden[1] Hospitals, London*

Twenty-five women with advanced breast cancer (16 post- and 9 pre-menopausal) were treated with D Ser (Bu$^t$)$^6$ LHRH ethylamide (buserelin), a long acting analogue of gonadotrophin releasing hormone. Buserelin was given in divided dosages of either 600 or 1000 $\mu$g daily intranasally for up to 7 months. Only one premenopausal and 4/8 postmenopausal women had received prior hormonal therapy and responded. Oestrogen receptors were present and measured in 4/6 premenopausal and none of 3 postmenopausal women. Early minimal responses were observed in 1 postmenopausal and 2 premenopausal patients. Both premenopausal responses were in oestrogen receptor positive patients but the oestrogen receptor status of the postmenopausal women was unknown. None of the responding patients had received previous endocrine treatment. These results suggest that buserelin may provide a further nontoxic hormonal therapy for breast cancer in premenopausal women, but its significance in the treatment of postmenopausal patients has not been proven.

### Distal transposition of the caecum in the rat does not affect susceptibility to carcinogenesis

J.B. Rainey, M. Maeda & R.C.N. Williamson

*University Department of Surgery, Bristol Royal Infirmary, Bristol BS2 8HW*

Like its human counterpart the appendix, the rat caecum is relatively resistant to carcinogenesis, possibly because of its luminal environment. We therefore tested the effect of exposing caecal mucosa to the distal faecal stream in male Sprague-Dawley rats ($n=50$) given a selective intestinal carcinogen. The colon was transected at the pelvic brim and the caecum was inserted isoperistaltically between colo-caecal and caeco-rectal anastomoses ($n=30$). An ileo-colic anastomosis restored intestinal continuity. Controls ($n=20$) had transection and reanastomosis at equivalent points of the bowel plus caecotomy and resuture. Operations were performed 1 week after a 6-week course of azoxymethane injections (total dose $90\,mg\,kg^{-1}$ s.c.).

Caecal crypt cell production rate, as determined stathmokinetically at 28 weeks, was unaffected by transposition. No tumours developed in either transposed or orthotopic caecum apart from 3 suture-line tumours found at the caecotomy site in controls. The colonic tumour yield in controls ($1.4 \pm 0.3$ per rat:mean $\pm$ s.e.) matched that after transposition ($1.5 \pm 0.2$), but anastomotic tumours were commoner after transposition ($1.8 \pm 0.3$ *vs.* $0.8 \pm 0.3$: $P<0.05$).

Transposed caecum remains relatively resistant to carcinogenesis compared with distal colon. The composition of faeces passing through the caecum seems unimportant in maintaining this resistance, which may be more readily attributable to local epithelial mechanisms.

**The effect of colitis upon the colonic carcinogen dimethyl hydrazine in a rat model**

P.G. Davey, E. McGuinness, S. Nicol & K.G. Wormsley

*Depts of Therapeutics and Pathology, Ninewells Hospital and Medical School, Dundee DD1 9SY*

The object of the experiment was to see whether colitis increases the susceptibility of the rat to the colonic carcinogen dimethyl hydrazine (DMH). Colitis was induced in adult, male Sprague Dawley rats by intrarectal injection of 10% acetic acid (IRAA) through 3 cm of PE240 polythene tubing under ether anaesthesia. In preliminary experiments we showed that a consistent degree of histological inflammation was produced up to 12 cm from the anus. Three groups of 14 rats were studied: group A received IRAA then 20 weekly s.c. injections of $10\,mg$ DMH $kg^{-1}$ in EDTA base; group B received IRAA then 20 weekly s.c. injections of EDTA base alone; group C received IR saline then 20 DMH injections. Eight rats from group A were killed because of severe colitis; all colons were dilated and most had perforated. One rat in group B died with a jejunal tumour at 17 weeks. The remaining rats were sacrificed at 20 weeks. No metastatic lesions were found in any rat and group B rats had no colonic tumours. Adenocarcinomas were present in the colons of all group A rats and 11/13 group C rats; group A rats had significantly more tumours per colon (Wilcoxon test, $P<0.01$); there were a total of 25 tumours in the 6 group A rats and 19 in the 13 group C rats. The stage of the tumours was the same in both groups and most had penetrated the submucosa, one tumour in each group penetrated through the bowel wall. The tumours in group A were significantly more distal (Wilcoxon test, $P<0.01$) with most tumours occurring within 10 cm from the anus whereas in group C tumours were more common in the proximal and mid colon. We conclude that DMH injections increase the severity of colitis induced by acetic acid and that the presence of colitis alters both the number and distribution of DMH induced colon cancers in the rat.

**Intrarectal deoxycholate promotes experimental colorectal carcinogenesis in the rat without affecting the colonic bacterial population**

J.B. Rainey, R. Veale & R.C.N. Williamson

*University Depts Surgery & Microbiology, Bristol Royal Infirmary, Bristol BS2 8HW*

The case for bile acids as promoters of colorectal carcinogenesis rests on human epidemiological data and animal experiments. If direct exposure of colorectal mucosa to bile acids altered the composition of the faecal flora, itself implicated as a modulator of carcinogenesis, animal experiments might have limited relevance to human carcinogenesis. The promotional effect of sodium deoxycholate (SDC) was tested in male Sprague-Dawley rats ($n=70$) which had received a 6-week course of azoxymethane (total dose $90\,mg\,kg^{-1}$ s.c.). For the next 18 weeks 2 groups received thrice weekly intrarectal instillations of 1 ml 0.12 M SDC ($n=25$) or 1 ml N saline ($n=25$). Controls had no instillations ($n=20$). At sacrifice (28 weeks), colorectal tumour yield (controls $2.4 \pm 0.4$ per rat: mean $\pm$ s.e.) was unaffected by intrarectal saline ($2.8 \pm 0.5$) but was almost trebled by intrarectal SDC ($6.4 \pm 0.5$) $P<0.01$. SDC also increased mean colonic crypt depth by 9% compared with the other 2 groups ($P<0.001$). Other rats ($n=9$) received intrarectal SDC or saline or no instillations without carcinogen and were killed at 12 weeks. Total colonic bacterial counts showed no consistent differences between groups. Sodium deoxycholate strongly promotes experimental colorectal carcinogenesis. This effect is probably due to its tropic action on the mucosa and is not related to any concurrent change in bacterial flora.

**Studies on the metabolic activation of chrysene**

R.M. Hodgson, A. Weston & P.L. Grover

*Chester Beatty Laboratories, Institute of Cancer Research, Royal Cancer Hospital, Fulham Road, London SW3 6JB*

The polycyclic aromatic hydrocarbon chrysene is a weakly active carcinogen on mouse skin, but the dihydrodiol precursor of its "bay-region" diol-epoxide is more carcinogenic than the parent compound. Ether extracts of hamster embryo cell cultures and mouse skin *in vivo* treated with chrysene were examined for the presence of dihydrodiol metabolites by t.l.c. and h.p.l.c. In addition the chrysene-nucleoside adducts formed in cultured hamster embryo cells, a rat liver microsomal metabolizing system containing DNA and mouse skin *in vivo* treated with $^3$H-labelled chrysene were examined by Sephadex LH20 column chromatography and by h.p.l.c. on Zorbax ODS. All three possible dihydrodiols of chrysene were detected in hamster embryo cells and mouse skin treated with chrysene. A chrysene triol was also found in mouse skin extracts. In hamster embryo cells, the major DNA-adducts had chromatographic properties identical to those of adducts formed when the *anti* isomer of the "bay-region" diol-epoxide of chrysene reacts with DNA. Both guanosine- and adenosine-hydrocarbon adducts were detected. NMR studies on the guanosine adducts showed that the hydrocarbon is attached at the exocyclic amino group of guanine. In the rat-liver microsomal preparations and in mouse skin treated with $^3$H-chrysene, adducts having chromatographic properties identical to those formed when *anti*-chrysene-1,2-diol 3,4-oxide reacts with DNA were also detected. Additional adducts were also present, however, which differed in their chromatographic mobilities from synthetic DNA-chrysene adducts formed from *anti*-chrysene-1,2-diol 3,4-oxide. The results show that metabolic activation of chrysene occurs via this "bay-region" diol-epoxide but suggest that, in some biological systems, this is not the sole route for the metabolic activation of chrysene.

**The nitrosation of drugs under chemical and simulated gastric conditions**

P.N. Gillatt[1], R.J. Hart[1], C.L. Walters[1] & P.I. Reed[2]

[1]*Leatherhead Food Research Association, Leatherhead, Surrey,* [2]*Gastrointestinal Unit, Wexham Park Hospital, Slough*

Using the Nitrosation Assay Procedure (F. Coulston, The potential carcinogenicity of nitrosatable drugs (Norwood, 1980 *Ablex Publ. Co.*), 60 drugs have undergone nitrosation at pH 3.0 with a molar ratio of nitrite:drug of 4:1. Volatile N-nitrosamines formed have been determined individually by gas chromatography with a Thermal Energy Analyzer as selective detector. More complex N-nitroso compounds have been determined as a group using a chemiluminescence analyzer as Walters *et al.* (1978) (*Analyst*, **103,** 1127).

The great majority of nitrogenous drugs are susceptible to the action of nitrous acid with the production of volatile N-nitrosamines or more complex N-nitroso derivatives or both. The extents of formation have varied widely under standardised chemical conditions, the nitrosation of secondary amino drugs occurring generally more readily than secondary amides, tertiary amines, hydrazides and carbamates with the exception of aminopyrine and minocycline, both of which are tertiary amines giving rise rise to high yields of N-nitrosodimethylamine (NDMA).

Nitrosations are being extended to simulated gastric conditions so as to ascertain the likely contact with a N-nitroso compound(s), the vast majority of which are carcinogenic in experimental animals, of a person treated with the normal daily dose of a series of drugs. So far, the yields of N-nitroso compounds as a group have ranged from $10^2$–$10^4$ times less than those obtained under the conditions of the NAP test.

**An experimental animal model for human invasive bladder cancer**

R.M. Hicks, K. Nandra, J. Turton, N. Tomlinson, J. Gwynne, E. Chrysostomou & M. Pedrick

*School of Pathology, Middlesex Hospital Medical School, London W1P 7LD*

Human papillary cancer usually presents as a superficial, well-differentiated papillary lesion, controllable by local resection for many years, but once carcinoma in situ(cis) and/or flat invasive transitional cell carcinoma (tcc) develop, bladder cancer is an aggressive neoplasm, difficult to control and often fatal. Several rodent models are available for the superficial papillary disease but none give reliably high incidences of cis or invasive tcc. We report here the availability of a rodent model for cis and tcc, developed from that described by Becci *et al.* (*Cancer Res.*, 1981, **41,** 927). The female B6D2F1 mouse, dosed intragastrically with N-butyl-N-(4-hydroxybutyl) nitrosamine (BBN), develops cis which rapidly progresses to invasive tcc, which is histologically very comparable to the human disease. Tumour incidence is directly related, and the latent period inversely related, to the dose of BBN. The time-course of cancer development was monitored by sampling 5–8 mice per group at regular intervals. By 50 weeks after 30 mg BBN given weekly as 10 equal aliquots the cumulative incidence was 28%; after 15 mg BBN it was 7% but after 10 mg there were no carcinomas. The lesions progressed from cis to flat invasive P1 and P2 tcc, and then to poorly-differentiated P3 lesions in which cancer cells invaded the peritoneal surface. In some areas squamous metaplasia developed and in others the growth pattern was adenomatous. This model provides a valuable new tool with which to study the development, treatment and modulation of invasive bladder cancer.

### Comparison of polyclonal and monoclonal anti-EMA antibodies

E. Heyderman[1], G. Powell[1], T.C. Richardson[1], D.V. Chapman[1], J.L. Cordell[2] & D.Y. Mason[2]

[1]*Dept Histopathology, St Thomas Hospital, London, SE1 7EH,* [2]*Dept Haematology, John Radcliffe Hospital, Oxford*

The ubiquitous nature of an antigen, or group of antigens, termed Epethelial Membrane Antigen (EMA), was first reported to this association in 1978 (Heyderman *et al., Br. J. Cancer,* 1978, **39,** 473). It was localised by the immunoperoxidase technique in a variety of tissues using polyclonal antibodies raised against human milk fat globule membranes. These antibodies have since been used extensively for the diagnosis and differential diagnosis of a variety of malignant tumours. To increase specificity, rabbit antibodies have been affinity-purified on an agarose column (AffiGel 10, BioRad, Herts) to which a preparation of milk fat globule membranes had been bound. Yields from this purification step were small, and the antibodies could not readily be made available to other workers. Monoclonal antibodies can be produced in large quantities, so we have compared the results of staining 25 different neoplastic and non-neoplastic tissues with a polyclonal unpurified rabbit anti-EMA (Dr Ormerod, ICR, Sutton), our affinity-purified antibody, and two monoclonal hybridoma antibodies, MFGM2 (Seward Laboratory, London), and our own monoclonal, E29/68. The tumours included carcinomas of the breast, colon, lung, kidney, ovary, and skin, and a malignant teratoma of the testis with epithelial differentiation. The non-neoplastic specimens consisted of pituitary, pancreas, skin, placenta, lactating breast, tonsil and hyperplastic prostate. While the distribution of staining with the four antibodies was similar, the hydridoma antibodies produced a less extensive staining pattern, and retained the membrane staining of some plasma cells. A combination of the two monoclonals was also assessed.

### The heterogeneity of CEA expression in gastric carcinoma

F. Macdonald, M.S. Hockey, H. Stokes & J.W.L. Fielding

*Dept. Surgery, Queen Elizabeth Hospital, Birmingham*

Monoclonal antibodies to different epitopes of CEA were used in an indirect immunoperoxidase test to study CEA expression in primary gastric tumours and metastases and to evaluate CEA as a potential target for therapeutic or diagnostic agents. Ninety-three per cent of 216 primaries and 82% of 148 autologous metastases examined with one antibody (L11-285-14) specific for CEA were positive. There was good correlation between CEA status of the primaries and metastases. However, in patients where two or more nodes were examined, 20% had both positive and negative nodes. Individual primaries and metastases were also heterogenous for CEA expression. Serial sections of 14 primaries and 14 metastases were therefore examined with 2 further monoclonal antibodies to different epitopes of CEA and 2 polyclonal anti-CEA antibodies to determine whether this heterogeneity reflected differences in CEA expression in different populations of cells or the specificity of the monoclonal antibody. All tumours negative with one antibody were negative with all and *vice versa.* All antibodies reacted with the same tumour cells. While CEA remains the most potentially useful antigen in gastric carcinoma, this study has demonstrated some possible limiting factors in both follow-up radioimmunolocalisation and drug targetting, even using a monoclonal "cocktail".

**Studies of WGA-binding proteins of metastatic tumour cells, macrophages and other cell types**

W.-S. Chan, A. Jackson & G.A. Turner

*University Dept. Clinical Biochemistry & Metabolic Medicine, Royal Victoria Infirmary, Newcastle upon Tyne NE1 4LP*

We have demonstrated (Chan *et al.*, 1982, *Br. J. Cancer*, **46**, 474) higher Wheat Germ Agglutinin (WGA) binding to glycoproteins extracted from a s.c. transplanted hamster lymphosarcoma (1°) as compared to the binding to proteins from its liver metastases (2°). This difference has been further investigated by subjecting tumour cell suspensions to various cell separation techniques and the protein extracts from these subpopulations analysed by using electrophoresis and $^{125}$I-WGA labelling. For comparison, extracts of potential tumour host-cell infiltrators, in particular macrophages, have been examined. Macrophage-rich populations prepared from 1° tumours by using density gradients showed increased expression of WGA-binding proteins. Only certain types of non-tumour macrophages expressed WGA-binding patterns that were similar to the 1° tumour pattern. However, macrophage-depleted tumour cell populations isolated by density gradients or carbonyl iron treatment or a cell adhesion technique still showed substantial WGA-binding. After further purification of these cell suspensions using unit gravity separation, the WGA-binding pattern was very similar in all fractions irrespective of its composition. Some of the high content of WGA-binding proteins in this 1° tumour, therefore, can be accounted for by the presence of infiltrating macrophages (~9% total cell no.), but tumour cells also seem to express very similar surface glycoproteins. Metastatic cells express these proteins very weakly; a reason for this difference awaits further investigation.

**Studies on serum galactosyl transferase in cancer patients**

P.A. Light, K. Howe, A.W. Preece, V.L. Barley & K.A. Walter

*Research Unit, Radiotherapy and Oncology Centre, Bristol BS2 8ED*

The activity of the enzyme galactosyl transferase (GT) has been reported to be elevated in the serum of patients with certain types of cancer. We have measured GT activity in serum of 305 patients with cancer (85% ca breast) and in 92 other subjects without malignant disease. The mean values of these groups were $31.0 \pm 27.3$ and $21.5 \pm 7.2$ units respectively. In the cancer group, of the 167 patients who had GT values above that of the control group, 92% had clinically active disease. However, in the remainder with low or normal values there were at least 30% with active disease. Serial samples taken during several months from ten patients showed close correlation with disease activity in 6 patients, but apparently little correlation in the remainder. Analogous studies using the L2C tumour in guinea pigs showed that serum GT activity correlated precisely with tumour burden. It has been suggested that the isoenzyme GT2 may be a more specific marker of malignant growth. However, we have not been able to confirm this observation. If the technique for measuring GT activity could be simplified then the assay might be a worthwhile additional test in monitoring progress of some patients. However, the present combination of a very time-consuming assay and the uncertainty in recognizing false negative results restricts its usefulness until improvements in techniques can be introduced.

**Viability studies on exfoliated colorectal cancer cells recovered from sites of intestinal transection and isolated on Nycodenz columns**

H.C. Umpleby, B. Fermor, M.O. Symes & R.C.N. Williamson

*University Department of Surgery, Royal Infirmary, Bristol*

Exfoliated colorectal cancer cells are reported to be non viable and therefore an unlikely cause of suture-line recurrence recurrence following resection of large bowel tumours (Rosenberg *et al.* (1978), *Br. J. Surg.*, **65**, 188). The presence and viability of exfoliated colorectal cancer cells at the proximal and distal sites of transection were investigated in 30 freshly-resected tumour specimens. Clamps were applied 3 cm from the transected bowel ends. Irrigation with tissue culture medium was performed on all proximal ends and on 25 distal ends. There were 5 cases of abdomino-perineal

| | Cases viable tumour cells | Trypan blue exclusion No. viable tumour cells x $10^5$ (median + range) | Median % viability | Fluorescence (no. cases) | Distance (cm) from tumour (median + range) |
|---|---|---|---|---|---|
| Proximal | 17 | 0.55(0.25–12.5) | 92.5 | 15 | 10.8(4–35) |
| Distal | 21 | 1.92(0.25–22) | 79.3 | 21 | 7.5(3–20) |

resection and 9 of right hemicolectomy. Tumour cells were isolated on Nycodenz density centrifugation columns (Umpleby *et al.* (1983), *Br. J. Cancer,* **48,** 127), and viability was determined by exclusion of the supravital stain trypan blue, and by hydrolysis of fluorescein diacetate to produce detectable fluorescence.

In 5 cases viable tumour cells were isolated from the ileum. Independent cytological examination confirmed tumour cells in all cases demonstrating viable cells. Large numbers of viable cancer cells are shed into the intestinal lumen and could therefore implant on the freshly created anastomosis.

### Autologous bone marrow transplantation contributes to haemopoietic recovery in children with solid tumours treated with high dose melphalan

J.E. Kingston[1], J. Pritchard[2], J.S. Malpas[1] & T.J. McElwain[3]

[1]*St Bartholomew's,* [2]*Great Ormond Street &* [3]*Royal Marsden Hospitals, London*

During a 5 year period 65 children with advanced stage malignant solid tumours have been treated with high dose melphalan (100–220 mg m$^{-2}$) combined with non cryopreserved autologous marrow transplantation. Forty-five of the children were given a "priming" dose of cyclophosphamide (300 mg m$^{-2}$) one week before treatment with melphalan. All children developed profound neutropenia and thrombocytopenia following high dose melphalan; the nadir of the neutrophil and platelet counts occurring at median intervals of 7 and 10 days respectively. The duration of neutropenia (median 11 days) and the duration of thrombocytopenia (median 18 days) did not appear to be affected by the dose of melphalan, suggesting that the initial recovery of the peripheral blood count results from the engrafted autologous marrow. There was a highly significant negative correlation between the nucleated cell count of the reinfused marrow and the period of neutropenia ($P<0.001$) and a significant negative correlation between the nucleated cell count and the period of thrombocytopenia ($P<0.01$). We conclude therefore, that autologous marrow transplantation accelerates haemopoietic recovery in children with solid tumours treated with high dose melphalan.

### Depressed natural killer activity in patients with malignant lymphoma

F. Healy, R.C. Rees & R.W. Hancock

*Depts Virology and Medicine, University of Sheffield*

The Natural Killer (NK) activity of peripheral blood lymphocytes (PBL) and whole blood from patients with malignant lymphoma, prior to treatment, was found to be significantly depressed when compared with controls. This is consistent with an intrinsic defect in the cell-mediated immune system, which is commonly found in these patients. A positive correlation was observed between the level of natural killing of PBL from HD patients and the age of the patients, although this was not seen with non-HD patients or with controls. However, among non-HD patients a positive correlation was observed between NK activity and the stage of disease, i.e., stage III and IV patients had significantly higher levels of NK activity than stage I and II patients. There was no correlation between the level of NK activity of lymphoma patients and either the histological type of disease or the sex of patients. NK activity of PBL from the majority of patients tested (12/18) could be boosted by pre-incubation with interferon and similarly the activity of whole blood samples from 7/10 patients was boosted by interferon. Using the monoclonal antibody Leu-7 as an NK marker, it was shown that the number of cells expressing this antigen in the peripheral blood of non-Hodgkin's patients was within the normal range, although expression of Leu-7 did not correlate with NK activity. Preliminary studies suggest that the number of Leu-7$^+$ cells in HD patients is also in the normal range.

### Depressed natural killer cell activity in blood draining digestive tract tumours in man

R.C. Rees, K. Rogers & C.D.M. Griffith

*Depts Virology and Surgery, The University of Sheffield Medical School*

The functional status of natural killer (NK) cell activity was assessed in patients undergoing surgery for digestive tract carcinoma or benign conditions. The level of natural killing was assessed in peripheral venous blood, the aortic blood supply to the tumour, and the tumour draining venous blood. NK cell activity of peripheral venous blood taken from cancer patients was significantly depressed compared with benign controls (pre-operative blood samples). In addition, assessment of the activity present in the blood draining the primary tumour site was shown to be significantly less than the activity present in the aortic blood suggesting that lymphocyte function is influenced as a result of passage through the tumour site. Plasma samples from either the tumour venous blood supply, or the peripheral venous supply were assessed for their effect on natural killing, mediated by peripheral blood lymphocytes from normal individuals. Blood plasma recovered from tumour draining blood caused significant ($P=0.01$) inhibition of NK cell killing of K562 targets in the majority of tests performed, whereas the corresponding peripheral venous blood from the same patients was only inhibitory in 25 per cent of cases. There was no significant change in the total number of blood lymphocytes before and after passage through the tumour (aortic versus tumour-venous blood lymphocytes) or in total white blood cell counts. Fluorescent staining with monoclonal antibody against lymphocyte surface markers also failed to reveal significant alterations in the level of T-lymphocytes and Leu-7 (NK marker) positive lymphocytes across the tumour. This study infers a role for soluble factors released from the primary tumour mass, in mediating depression of NK cell function.

### Characterisation of mononuclear cells of infiltrates in human colorectal carcinomas using monoclonal antibodies

H.C. Umpleby, D. Heinemann, M.O. Symes & R.C.N. Williamson

*Dept. of Surgery, University of Bristol, Bristol*

Serial frozen sections were prepared from 11 colorectal carcinomas and from adjacent normal bowel in 7 of these cases. The mononuclear cell infiltrates were stained by indirect immunoperoxidase using a panel of mouse monoclonal antibodies to human leucocyte antigens. The degree of infiltration was graded 5 (heavy), 4 (moderate). 3 (few), 2 (occasional) and 1 (nil). Assessment was performed by two independent observers. All sections of carcinoma save one showed a heavy or moderate infiltrate with leucocytes (anti HLe-1) among which T cells (UCHT-1) were predominant. There was an excess (graded 3–4) of suppressor/cytotoxic T cells (UCHT 4) over helper/inducer T cells (OKT 4) (graded 1–2) in 8 tumours. The T cells were not all activated as indicated by the reduced number (graded 2–3) staining with HLA-DR antibody (OK 1aI). No NK cells were found in 2 tumours tested (HNK-1). The findings for adjacent normal bowel were similar except that suppressor/cytoxic T cells did not predominate over helper/inducer T cells. The excess of cytotoxic T cells in tumour tissue is consistent with the cytotoxicity of tumour infiltrating lymphocytes for autoplastic tumour cells (Hutchison *et al.*, (1981), *Br. J. Cancer,* **44,** 396) and the positive relationship between lymphoid infiltration in tumours and a favourable prognosis (Underwood, (1974), *Br. J. Cancer,* **30,** 538; Hutchinson *et al.*, (1983), *J. Exp. Clin. Cancer Res.*, **2,** 161).

### Transplantability of tumour cell clones before and after passage in immunodeficient hosts

M.F.A. Woodruff[1], B.A. Hodson[2] & J.A. Ansell[1]

[1]*MRC Clinical and Population Cytogenetics Unit, Edinburgh, and* [2]*Department of Zoology, University of Edinburgh*

Cloned cell lines from strongly immunogenic methylcholanthrene-induced murine fibrosarcomas often fail to grow when transplanted s.c. directly ($10^6$–$2\times10^6$ viable cells) to normal syngeneic hosts. Uncloned populations from the primary tumour transplant readily but long-cultured uncloned lines may fail to do so. Cloned and uncloned cell lines grow readily in thymectomized, heavily irradiated (730 rad) mice protected with cytosine arabinoside, sublethally irradiated (495 rad) and nude mice, and after such passage usually grow in normal mice. Studies with one particular unpassaged clone have shown that it fails to grow in normal mice over a wide dose range ($10^3$–$10^7$ cells), when mixed with untreated or irradiated peritoneal exudate cells or cells of another clone, or when a passaged clone is transplanted elsewhere in the same host, but does grow in mice irradiated up to 24 (exceptionally 48) h after tumour inoculation. We postulate that cloned lines are vulnerable to the combined effect of mechanisms mediated by T cells and NK or related cells but escape if either component is missing. During passage in hosts deficient in T but not NK cells, a tumour cell population emerges which is NK cell resistant and can therefore grow in normal mice. To test this hypothesis we are studying the sensitivity of passaged and unpassaged clones to killing by isogenic spleen cells *in vitro*, and the transplantability of clones passaged in hosts deficient in both T and NK cells.

**Migration of human T and B lymphocytes in three-dimensional collagen matrices**

W. Shiu & S. Schor

*CRC Department of Medical Oncology, Christie Hospital & Holt Radium Institute, Manchester*

We have previously presented quantitative data regarding the migration of unfractionated human peripheral blood lymphocytes into three-dimensional collagen matrices (Schor *et al., J. Cell Biol.,* (1983), **96,** 1089). Using this experimental system, we now present data regarding the comparative migratory behaviour of fractionated T and B lymphocyte subsets. Enriched populations of T and B lymphocytes were prepared by standard rosetting techniques using sheep red blood cells. The cellular constituents of the enriched populations were estimated by immunofluorescent microscopy using BA1 (Hybritech) and UCHT1 (supplied by Dr P. Beverley) monoclonal antibodies; the enriched lymphocyte populations contained 90% T cells and 70% B cells respectively. When plated on the surface of the collagen gel matrix, T and B cells were observed to enter the collagen matrix at the same rate. Once within the collagen matrix, however, T cells migrated significantly faster than B cells. As previously shown for the unfractionated lymphocytes, the migration of both T and B cells was found to occur by a random walk mechanism.